# Engineered Sporopollenin Exine Capsules for Colon-Targeted Delivery and Antioxidant Therapy of Pogostemon Oil in Ulcerative Colitis

**DOI:** 10.3390/antiox15010116

**Published:** 2026-01-16

**Authors:** Jia Si, Shasha Dai, Huaiyu Su, Zhongjuan Ji, Cong Dong, Xinao Lyu, Shuhuan Lyu, Lin Chen, Jianwei Sun, Xiangqun Jin, Haiyan Li

**Affiliations:** 1 Department of Pharmacy, Jilin University, Changchun 130021, China; jiasi23@mails.jlu.edu.cn (J.S.); daiss24@mails.jlu.edu.cn (S.D.); suhy24@mails.jlu.edu.cn (H.S.); jizj23@mails.jlu.edu.cn (Z.J.); dongcong23@mails.jlu.edu.cn (C.D.); lvxa24@mails.jlu.edu.cn (X.L.); lvsh25@mails.jlu.edu.cn (S.L.); chenlin25@mails.jlu.edu.cn (L.C.); sunjw25@mails.jlu.edu.cn (J.S.); 2 Department of Pharmacy, Changchun University of Chinese Medicine, Changchun 130021, China

**Keywords:** sporopollenin exine capsules, Pogostemon oil, ulcerative colitis, antioxidant

## Abstract

Ulcerative colitis (UC) is an inflammatory bowel disease associated with oxidative stress. Pogostemon oil (PO) exhibits potent antioxidant and anti-inflammatory activities but is limited by high volatility and poor gastrointestinal stability. In this study, sporopollenin exine capsules (SECs) were engineered as natural micro-carriers for PO, achieving efficient encapsulation (η > 69%) and a high adsorption capacity (27.64 g/g). A pH-sensitive calcium alginate shell was subsequently applied to construct colon-targeted microspheres (Ca-Alg@PO-SECs). The resulting system improved the thermal and photostability of PO. In vitro dissolution assays confirmed the system’s pH-responsiveness, maintaining integrity under simulated gastric conditions while enabling localized release at intestinal pH. In a DSS-induced acute UC mouse model, Ca-Alg@PO-SECs effectively alleviated clinical symptoms, as evidenced by improved body weight, colon length, and disease activity index. At the inflammatory level, the formulation modulated key cytokines (IL-1β, IL-6, and IL-10). Overall, Ca-Alg@PO-SECs provides a biocompatible, colon-targeted delivery strategy that preserves the bioactivity of essential oils and offers a promising preclinical approach for localized UC therapy.

## 1. Introduction

Ulcerative colitis (UC) is a chronic, relapsing, and non-specific inflammatory bowel disease (IBD) with a complex aetiology, involving immune dysregulation, impairment of the intestinal mucosal barrier, and excessive activation of oxidative stress responses [1]. A key driver of its pathogenesis is oxidative stress. In the inflamed colon, excessive reactive oxygen species (ROS) produced by immune and epithelial cells trigger lipid peroxidation and mitochondrial dysfunction.

Elevated ROS levels mediate significant cellular damage, particularly by triggering lipid peroxidation, oxidative DNA damage, and mitochondrial dysfunction. Concurrently, ROS persistently activate core inflammatory cascades such as NF-κB and MAPK, thereby driving chronic inflammation [2,3]. The main pathological features are persistent colonic mucosal inflammation, leading to ulcer formation [4,5]. Conventional therapies such as corticosteroids, aminosalicylates, and immunosuppressants reduce inflammation and alleviate symptoms by modulating immune pathways [6]. However, prolonged use often causes high relapse rates. Systemic exposure leads to adverse effects, and limited targeting reduces therapeutic specificity [7,8,9]. As a result, developing safe, efficient, and natural antioxidant drug delivery systems is a key focus in current UC therapy research.

Natural bioactive compounds have recently gained attention as adjuvant treatments for IBD due to their multitarget activities, low toxicity, and excellent biocompatibility [10]. Pogostemon oil (PO), a key volatile constituent of traditional Chinese medicine, is notable for its high content of pharmacologically active terpenoids. These terpenoids, especially patchouli alcohol and pogostone, contribute to its medicinal value [11,12]. Modern pharmacological studies have demonstrated that the various bioactive constituents of PO exert therapeutic effects through multiple mechanisms. These include inhibiting the release of pro-inflammatory cytokines [13,14], attenuating oxidative stress [15], and modulating the gut microbiota composition [16]. PO and its active constituents exhibit robust antioxidant activity. This is a principal therapeutic mechanism in the management of colitis. However, PO is highly hydrophobic and volatile. It is susceptible to gastric acid degradation and volatilization during oral administration [17]. As a result, these properties hinder its ability to achieve and maintain effective concentrations in the colon.

Researchers are now focusing on porous microsphere carrier systems, made from natural materials, to address the stability challenges associated with delivering natural oily drugs [18]. Among these, sporopollenin exine capsules (SECs) are derived from pollen but are processed to remove allergenic proteins, yielding a chemically inert and structurally robust biocarrier suitable for preclinical applications. They possess a unique porous microcavity structure, exhibit exceptional chemical stability, are resistant to acid–base reactions, and demonstrate excellent biocompatibility [19]. These structural attributes establish SECs as an ideal adsorptive matrix for oily drugs [20] and an effective physical barrier for volatile compounds [21], a capability demonstrated by previous studies on the encapsulation of various small-molecule drugs [22] and bioactive compounds [23]. However, adsorption performance is specifically influenced by the origin of the pollen and the acidolysis conditions. Although this variability is well recognized, a systematic and quantitative understanding of how SEC microstructure influences the adsorption capacity for oily drugs is still lacking. Moreover, conventional drug-loading methods, such as passive and vacuum adsorption [24], are inefficient and therefore unsuitable for encapsulating highly volatile drugs like PO. Furthermore, standalone SECs also fail to deliver the precise colonic targeting and controlled release required for UC therapy.

This study developed a novel composite carrier system that combines high loading efficiency, colonic targeting, and sustained release to enhance the stability and delivery of PO. The process began with a structure–property analysis, which identified sunflower pollen-derived SECs as the optimal core carrier for loading oily drugs. Next, a selective adsorption technique was optimized. This achieved high PO loading and efficient encapsulation, further improving its stability and drug-loading performance. For colonic-targeted release, calcium alginate (Ca-Alg) was used to encapsulate PO-SECs, forming a core–shell composite carrier (Ca-Alg@PO-SECs). The Ca-Alg shell exhibits exceptional light and temperature isolation, effectively suppressing oxidative degradation of the active PO components induced by external factors. Within the colonic environment, the shell undergoes pH- and enzyme-responsive swelling and degradation, thereby enhancing gastric acid tolerance and facilitating site-specific, sustained PO release at inflammatory lesions.

For colonic-targeted release, calcium alginate (Ca-Alg) was used to encapsulate PO-SECs, forming a core–shell composite carrier (Ca-Alg@PO-SECs). The Ca-Alg shell offers exceptional light and temperature isolation. It effectively suppresses oxidative degradation of the active PO components induced by external factors. Within the colonic environment, the shell undergoes pH- and enzyme-responsive swelling and degradation. This enhances gastric acid tolerance and facilitates site-specific, sustained PO release at inflammatory lesions.

In summary, this study optimized the preparation and loading processes of SECs, clarifying the structural regulation and adsorption mechanisms for oily drugs. Building on this foundation, we rationally designed a novel natural drug delivery system with high loading capacity, structural stability, and colon-targeted sustained-release properties. We systematically evaluated its release behavior and therapeutic performance through in vitro release studies and pharmacodynamic assessment in a DSS-induced UC mouse model. This engineered strategy provides experimental support for targeted intervention in ulcerative colitis and demonstrates the potential to alleviate oxidative stress driven by excessive reactive oxygen species during disease progression.

## 2. Materials and Methods

### 2.1. Materials

Rapeseed pollen (Jiannan Honey Shop, Binjiang Subdistrict Office, Nanbu County, China), tea pollen and sunflower pollen (Changge Spring Blossom Bee Industry Co., Ltd., Changge, China), and rose flower pollen (Xinzhou Wutai Mountain Bee Industry Co., Ltd., Xinzhou, China) were obtained for this study.

Acetonitrile, glycerol, anhydrous ethanol, phosphoric acid, and petroleum ether (all analytical grade, 85%, *v*/*v*), as well as sodium alginate and calcium chloride (Jilin Jintai Chemical Glass Co., Ltd., Jilin, China), were used as chemical reagents.

Simulated gastric fluid (SGF), simulated intestinal fluid (SIF), PBS, and mesalazine (5-aminosalicylic acid, 5-ASA)were obtained from Dalian Meilun Biotechnology Co., Ltd., Dalian, China, and used as the positive drug in the ulcerative colitis model.

Dextran sulfate sodium (DSS), BSA standard solution, and patchouli oil (extracted from Pogostemon cablin (Blanco) Benth)were purchased from Shanghai McLean Chemical Technology Co., Ltd., Shanghai, China. Double-distilled water was employed throughout the experiments. All reagents and chemicals were of analytical grade or chromatographic grade as specified.

### 2.2. Isolation and Processing of SECs

SECs were prepared through chemical extraction and removal steps. During this process, intracellular contents, lipids, hydrophilic impurities, and cellulose/pectin layers were sequentially eliminated. This yielded the intact sporopollenin exine framework.

#### 2.2.1. Extraction of Pollen

Sample pretreatment: Initially, the hydrophilic components and insoluble impurities are removed by dissolving the pollen in double-distilled water, followed by filtration through an 180 μm sieve. Subsequently, vacuum filtration was continued until the filtrate became clear, transparent, and colorless. Finally, the dried pollen was weighed and suspended in deionized water at a 1:5 (*w*/*v*) ratio. The resulting suspension was then filtered through an 180 μm sieve to eliminate any residual coarse impurities. Immediately thereafter, vacuum filtration was performed, and the filter cake was washed repeatedly with deionized water until the filtrate became clear. Subsequently, the resulting filter cake was transferred to an oven (or a forced-air drying oven) maintained at 60 °C and dried for 24 h.

Defatting Treatment: Initially, the dried pollen material was introduced into absolute ethanol at a ratio of 1:10 (*w*/*v*) and subjected to reflux at 90 °C for 1 h. Subsequently, the suspension was washed and filtered using absolute ethanol until the resulting filtrate became colorless. Finally, the resulting filter cake was transferred to an oven maintained at 60 °C and dried for 24 h, thereby yielding the defatted pollen material.

#### 2.2.2. Acid Hydrolysis Preparation of SECs

Protein Removal from Pollen (Deproteinization): Following the procedure reported by [25], 5 g of defatted pollen was accurately weighed and suspended in 100 mL of concentrated phosphoric acid (85 wt%). The mixture was stirred vigorously at 70 °C using a thermostatically controlled magnetic stirrer.

In this study, the acid hydrolysis (stirring) time was systematically varied from 6 to 24 h and designated as the principal independent variable, while the protein removal rate from sporopollenin and the particle size of the resulting products were used as the key evaluation metrics to optimize the preparation conditions.

Upon completion of the acid hydrolysis treatment, the resulting product was recovered by vacuum filtration. The solid residue was subsequently rinsed several times with hot water, then washed repeatedly with deionized water until the filtrate became neutral, colorless, and transparent. Finally, the obtained SECs were dried in a forced-air oven at 60 °C for 24 h prior to further characterization.

Preparation of Test Solutions: The entirety of the filtrate generated from the aforementioned acid hydrolysis step was collected and quantitatively diluted to a final volume of 1000 mL in a volumetric flask, thereby preparing the test solution ( $C_{\mathrm{Test}}$) for determining the content of protein eliminated during the acidolysis process.

#### 2.2.3. Method for Protein Quantification

(1) Preparation of Total Pollen Protein Extract ($C_{\mathrm{Total}}$): Following the procedure, an accurate mass of 5 g of pollen was precisely weighed and subsequently frozen at −18 °C for 24 h. Subsequently, ultrapure water was added at a ratio of 35 mL/g for the extraction. During the extraction process, the mixture was first heated in a water bath at 65 °C, followed by sonication for 60 min. Finally, the supernatant was collected via centrifugation and subsequently made up to 100 mL in a volumetric flask, thus yielding the total pollen protein extract ($C_{\mathrm{Total}}$). Acid-hydrolyzed samples were neutralized and washed, and protein-free controls showed no biuret interference.

(2) Methodology for Protein Content Determination: Precise volumes of the Bovine Serum Albumin (BSA) standard solution (5 mg/mL), specifically 0, 0.05, 0.1, 0.2, 0.4, and 0.8 mL, were accurately pipetted. Each solution was brought up to a total volume of 1.0 mL with distilled water, followed by the precise addition of 4.0 mL of Biuret reagent. After thorough mixing, the solutions were allowed to stand at room temperature (>20 °C) for 30 min to ensure complete reaction.

Subsequently, the absorbance of the solutions was measured at 540 nm, utilizing the corresponding reagent as the blank reference. The obtained absorbance values were then subjected to linear regression analysis to construct the standard curve and derive its governing equation.

An accurate volume of 1.0 mL of the test solution ($C_{\mathrm{Test}}$) was precisely pipetted; the protein content of the solution and Protein Removal Rate were calculated based on the following formula.

Formula for Protein Content Calculation:

$$Protein\;Content=\frac{(A_{X}-A-A_{0})\times C_{1}\times V_{1}}{{(A}_{1}-A_{0})\times f}$$

In the formula, $A_{0}$ represents the absorbance of the biuret reagent, A is the absorbance of the blank sample, $A_{X}$ is the absorbance of the test sample $C_{1}$ is the concentration of the standard solution, $V_{1}$ is the volume of the standard solution, $A_{1}$ is the absorbance of the diluted standard solution, and f is the dilution factor (10).

Formula for Protein Removal Rate Calculation:

$$Protein\;Removal\;Rate=\frac{M_{sample}\times 10}{M_{total}}\times 100\%$$

In the formula, $M_{total}$ represents the total protein content in the different pollen protein test solutions, and $M_{sample}$ represents the protein content in the total pollen protein extract.

### 2.3. Characterization

#### 2.3.1. Microscopic Imaging and Particle Size Analysis

Samples were placed under an OLYMPUS optical microscope at 100× magnification (Tokyo, Japan). For particle size analysis, each sample was first suspended and uniformly dispersed in deionized water. A drop of this suspension was then transferred onto a clean glass slide. More than 200 particles were randomly selected from at least three independent fields, and their equivalent diameters were measured using ImageJ software (v.1.54r) to determine the mean particle size ($D_{avg}$).

#### 2.3.2. Scanning Electron Microscopy (SEM) Analysis

The surface morphology of all samples was examined using a scanning electron microscope (ZEISS EVO 18, Oberkochen, Germany) at an accelerating voltage of 20.0 kV. For morphology comparison before and after chemical treatment, specimens were mounted on conductive carbon tape and coated with Au/Pd using a sputter coater (15 mA, 100 s, 27 mm working distance) to ensure high-resolution imaging.

#### 2.3.3. Fourier Transform Infrared Spectroscopy (FTIR) Analysis

The FTIR spectra of natural pollen and sporopollenin samples treated for different acidolysis durations were recorded using a spectrometer (Thermo Fisher Scientific iS10, Waltham, MA, USA with the KBr pellet technique. All spectra were collected at a resolution of 4 cm^−1^ with 32 scans over the range of 4000–400 cm^−1^ to characterize the variations in functional groups and chemical composition of sporopollenin before and after chemical treatment.

#### 2.3.4. Distribution of Sporopollenin in Different Solvents

We added approximately 100 mg of sample to 10 mL of deionized water, soybean oil, absolute ethanol, and glycerol. We immediately recorded the initial dispersion state. To assess stability, we let the samples stand at room temperature for 30 min, then recorded whether they sedimented or dispersed. We used these observations to evaluate the macroscopic compatibility and dispersion stability of SECs in solvents of different polarity.

#### 2.3.5. Wettability Evaluation

We evenly spread and compressed the SEC’s powder to form a smooth pellet. Using deionized water droplets, we measured the water contact angle (WCA) at room temperature. For each sample, we measured at five positions and calculated the mean to quantify changes in surface wettability before and after preparation.

### 2.4. Evaluation of the Oil Adsorption Capacity of SECs

This section aims to evaluate the adsorption characteristics of different types of SECs toward a model oil (pure soybean oil) and oil–water emulsion systems using standardized procedures.

#### 2.4.1. Evaluation Method for Adsorption Capacity and Efficiency

The adsorption capacity was determined gravimetrically. The dry mass of the initial ($m_{0}$), the mass at time t ($m_{t}$) and the mass after adsorption equilibrium ($m_{\mathrm{eq}}$) were measured, and the adsorption capacity ($Q_{t}\;or\;Q_{e}$) and adsorption efficiency (η) were subsequently calculated.

$$m_{\mathrm{adsorbed},t}=m_{t}-m_{0}$$

$$m_{\mathrm{adsorbed}}=m_{\mathrm{eq}}-m_{0}$$

$$Q_{t}(g/g)=\frac{m_{\mathrm{adsorbed},t}}{m_{\mathrm{SECs}}}$$

$$Q_{e}(g/g)=\frac{m_{\mathrm{adsorbed}}}{m_{\mathrm{SECs}}}$$

$$\eta (\%)=\frac{m_{\mathrm{adsorbed}}/m_{\mathrm{adsorbed},t}}{m_{\mathrm{oil}\text{\_}\mathrm{total}}}\times 100$$

$m_{\mathrm{eq}}$ represents the mass of sporopollenin after adsorption equilibrium (g); $m_{t}\;is\;the\;mass\;at\;adsorption\;time\;t(g);\;m_{0}$ is the initial mass of SECs (g); $m_{\mathrm{oil}\text{\_}\mathrm{total}}$ denotes the initial mass of the oil sample (g); $m_{\mathrm{adsorbed}}$ refers to the amount of oil adsorbed at time t (g); $m_{\mathrm{adsorbed},t}$ is the amount of oil adsorbed at time t (g) and $m_{\mathrm{SECs}}$ corresponds to the mass of the SECs adsorbent (g).

#### 2.4.2. Determination of Maximum Oil Absorption Capacity of SECs (Pure Oil Saturation Method)

Pure soybean oil was used as a model oil to evaluate the maximum oil absorption capacity ($Q_{e,\max}$) of SECs derived from different pollen species. Precisely weighed 100 mg of dried SECs were placed in nonwoven fabric bags and immersed in an excess amount of pure soybean oil until adsorption equilibrium was reached. After adsorption, the oil-loaded samples were removed from the oil phase and transferred into centrifuge tubes. Non-adsorbed oil droplets adhering to the surface of the SECs were removed by standardized low-speed centrifugation (1000× *g*, 15 min). The maximum oil absorption capacity ($Q_{e,\max}$) of sporopollenin was calculated according to the method described in Section 2.4.1

#### 2.4.3. Evaluation of Adsorption Capacity of SECs in Organic Solvents

A standardized procedure was employed to systematically evaluate the adsorption performance of SECs toward organic solvents with varying polarity, including ethanol, acetone, and n-hexane. Precisely 100 mg of dried SECs were added into 20 mL of pure solvent and shaken on a thermostatic oscillator until adsorption equilibrium was achieved. After equilibration, the samples were separated by high-speed centrifugation (3000× *g*, 5 min). The solvent-loaded SECs were immediately transferred to a vacuum desiccator (60 °C, −0.1 MPa) and dried to a constant weight.

Finally, the adsorption capacity of SECs was calculated according to the method described in Section 2.4.1, providing a quantitative assessment of their affinity and adsorption capacity toward different organic solvents.

#### 2.4.4. Investigation of the Equilibrium Adsorption Performance of SECs Toward Soybean Oil in Oil–Water Emulsions

This study systematically investigated the effects of three single factors. These include SEC dosage, adsorption time, and initial soybean oil concentration. The study evaluated their impact on the adsorption efficiency (η) and adsorption capacity ($Q_{e}$) of SECs.

In the experiment, an appropriate amount of soybean oil was precisely weighed and mixed with 25 mL of deionized water, followed by stirring with a turbine agitator for 10 min to prepare soybean oil emulsions with designated concentrations. Subsequently, a specified amount of SECs was added and stirred for another 10 min to ensure homogeneity, then continuously agitated under controlled conditions until adsorption equilibrium was achieved.

After the adsorption process, the oil-loaded SECs were separated by vacuum filtration and washed twice with deionized water to remove any residual oil. The obtained samples were immediately placed in a vacuum desiccator at 60 °C and −0.1 MPa until a constant weight was reached.

Finally, the adsorption efficiency (η) and adsorption capacity ($Q_{e}$) were calculated according to the method described in Section 2.4.1, providing a quantitative evaluation of the SECs adsorption performance toward the oil components within the emulsion system.

### 2.5. Adsorption Performance of SECs Toward Pogostemon Oil

To evaluate the adsorption characteristics of SECs toward a representative oily drug (PO), three methods were used: vacuum adsorption, passive adsorption, and selective adsorption.

High-performance liquid chromatography (HPLC) was used to determine the adsorption efficiency (η) and maximum adsorption capacity (M), thereby assessing the adsorption behavior of SECs under vacuum adsorption and passive adsorption.

In addition, the selective adsorption of SECs toward PO was analyzed using a gravimetric method, by calculating the adsorption efficiency (η) and maximum adsorption capacity (M), providing a quantitative understanding of their adsorption mechanism and affinity.

#### 2.5.1. Adsorption Evaluation Criteria

(1) High-Performance Liquid Chromatography (HPLC) Method.

Chromatographic Conditions and Sample Preparation.

Chromatographic Conditions and Solution Preparation.

Chromatographic column: Amethyst C18-H column (4.6 mm × 250 mm, 5 μm).

Mobile phase: acetonitrile: 0.2% phosphoric acid solution = 60:40 with a flow rate of 1.0 mL/min.

The column temperature: 30 °C,

The detection wavelength: 310 nm.

Injection volume: 10 μL.

After the adsorption process, the supernatant was collected and filtered through a 0.22 μm organic membrane. The amount of oil adsorbed at time t ($m_{\mathrm{adsorbed},t}$) was calculated based on the residual PO concentration in the solution determined, according to the external standard method. The oil adsorption amount per unit solution volume ($M_{t}$) and adsorption efficiency (η) were calculated as follows:

$$m_{\mathrm{adsorbed},t}=m_{\mathrm{oil}\text{\_}\mathrm{total}}-\frac{A_{\mathrm{Test}}}{A_{0}}\times C_{0}\times V_{\mathrm{Test}}$$

$$M_{t}\left(g/L\right)=\frac{m_{\mathrm{adsorbed},t}}{V_{\mathrm{Test}}}\times 1000$$

$$\eta (\%)=\frac{m_{\mathrm{adsorbed},t}}{m_{\mathrm{oil}\text{\_}\mathrm{total}}}\times 100$$

$A_{0}$ is the peak area of the standard solution; $C_{0}$ is the concentration of the standard solution (g·L^−1^); $A_{\mathrm{Test}}$ is the peak area of the test solution at adsorption time t; $V_{\mathrm{Test}}$ is the volume of the adsorption solution (L); $m_{\mathrm{adsorbed},t}$ is the mass of oil adsorbed by the SECs at time t (g) and $m_{\mathrm{oil}\text{\_}\mathrm{total}}$ is the initial total mass of oil added to the system (g).

(2) Gravimetric Method.

The adsorption capacity and efficiency of SECs toward oily substances were determined by measuring the mass difference before and after adsorption. The dried SECs sample was weighed as $m_{0}$, the mass at time t ($m_{t}$) and after reaching adsorption equilibrium or saturation, the sample was removed, gently wiped to remove non-adsorbed oil, and reweighed as $m_{\mathrm{eq}}$. The maximum adsorption capacity (M) and adsorption efficiency (η) were calculated as follows:

$$m_{\mathrm{adsorbed},t}=m_{t}-m_{0}$$

$$m_{\mathrm{adsorbed}}\left(g\right)=m_{\mathrm{eq}}-m_{0}$$

$$M_{t}\left(g/L\right)=\frac{m_{\mathrm{adsorbed},t}}{V_{\mathrm{Test}}}\times 1000$$

$$\eta (\%)=\frac{m_{\mathrm{adsorbed},t}}{m_{\mathrm{oil}\text{\_}\mathrm{total}}}\times 100$$

$m_{\mathrm{eq}}$ is the mass of SECs after adsorption equilibrium (g); $m_{t}\;is\;the\;mass\;at\;adsorption\;time\;t(g)$; $m_{0}$ is the initial mass of SECs (g); $m_{\mathrm{adsorbed}}$ represents the amount of oil adsorbed at time t (g); $m_{\mathrm{oil}\text{\_}\mathrm{total}}$ is the total initial mass of oil used in the experiment (g); and M refers to the maximum amount of oil that can be adsorbed per liter of solution (g/L).

#### 2.5.2. Passive Loading Technique

This study examined how SECs dosage, adsorption time, and PO concentration influence adsorption efficiency (η) and maximum adsorption capacity (M).

The passive loading technique involves co-incubating SECs with PO dissolved in ethanol at a controlled temperature, allowing the oil to spontaneously diffuse into and encapsulate the microcapsules. Briefly, we directly added a predetermined mass of SECs to the ethanolic PO solution and mixed it using a vortex mixer for 10 min. We then placed the resulting suspension on a thermostatic shaker and agitated it for a specified time to achieve passive adsorption. Once the adsorption equilibrium was reached, we filtered out the oil-loaded SECs and washed them with ethanol to remove surface-bound oil. We determined the adsorption performance using HPLC as described in Section 2.5.1. Finally, we freeze-dried the samples to a constant weight and stored them at −18 °C to prevent volatilization or degradation of the essential oil.

#### 2.5.3. Vacuum Loading Technique

We then used a single-factor experimental design to examine how SECs dosage, adsorption time, and PO concentration affect adsorption efficiency (η) and maximum adsorption capacity (M).

After applying the passive loading technique, which relies on diffusion at atmospheric pressure, we also employed the vacuum loading method to enhance the penetration and adsorption of PO molecules into the inner cavities of SECs under reduced pressure. Specifically, we first dissolved PO in ethanol to obtain a homogeneous solution. Next, we added a predetermined amount of SECs and mixed it using a vortex mixer for 10 min to ensure a uniform suspension. We transferred the mixture into a vacuum desiccator and maintained it under vacuum for a designated period to achieve adsorption equilibrium. Afterward, we used HPLC as described in Section 2.5.1 to determine adsorption efficiency and maximum adsorption capacity. We then freeze-dried and stored the oil-loaded SECs at −18 °C.

#### 2.5.4. Selective Adsorptive Loading Technique

We developed a novel selective adsorption method that improves the loading efficiency of hydrophobic active compounds into sporopollenin. Unlike other techniques, this method uses the synergistic effects of hydrophobic interactions and capillary adsorption between the sporopollenin surface and PO molecules to achieve selective and efficient encapsulation.

In this process, we dispersed PO in distilled water under stirring to form a stable oil–water suspension. We added a predetermined amount of SECs and vortex-mixed for 10 min to facilitate adequate contact between the SECs and oil droplets. We then placed the mixture on a thermostatic shaker and agitated it until adsorption equilibrium occurred. We filtered the oil-loaded SECs and washed them with ethanol to remove residual surface oil. We pre-froze, freeze-dried, and stored the collected microcapsules at −80 °C. We calculated adsorption efficiency (η) and maximum adsorption capacity (M) using the gravimetric method described in Section 2.5.1.

For the selective adsorption method, initial PO concentrations below 10 g/L were excluded from the study as the resulting mass changes were below the limit of quantitation (LOQ) for reliable gravimetric analysis.

### 2.6. Preparation of Ca-Alg@PO-SECs Composite Microspheres

We prepared Ca-Alg@PO-SECs composite microspheres using an emulsion–crosslinking technique.

Briefly, we thoroughly mixed 300 mg of PO-loaded sporopollenin capsules (PO-SECs) into 200 mL of 2.5% (*w*/*v*) sodium alginate (SA) solution to form a homogeneous aqueous phase. Next, we added this mixture dropwise to the oil phase, which consisted of Span-80 and Tween-80 blended at a 2:1 (*v*/*v*) ratio. We kept the aqueous-to-oil ratio at 1:1 (*v*/*v*) during addition, which took place under constant stirring. Emulsification was performed under continuous mechanical stirring at 600 rpm and 25 ± 1 °C for 3 h, resulting in a stable water-in-oil (W/O) emulsion.

Subsequently, a 2% (*w*/*v*) calcium chloride (CaCl_2_) solution was introduced dropwise at a controlled rate of approximately 1 mL·min^−1^ into the emulsion under constant stirring (600 rpm), initiating ionic crosslinking of the alginate droplets. The system was gently stirred for an additional 30 min at room temperature to ensure sufficient Ca^2+^-mediated gelation and formation of Ca-Alg-crosslinked microspheres. The stirring speed, temperature, and precise duration were monitored to ensure consistent microsphere formation.

After crosslinking, the resulting microspheres were collected by centrifugation (3000 rpm, 5 min), then washed them three times with anhydrous ethanol followed by three times with petroleum ether to remove residual oil and surfactants. We vacuum-filtered the purified microspheres and freeze-dried them to obtain dry Ca-Alg@PO-SECs composite microspheres. These were stored in a desiccator at 4 °C until further use.

### 2.7. Stability Evaluation of Microspheres

To assess the stability of sporopollenin and its combined microspheres (Ca-Alg@PO-SECs) under different conditions, we conducted stability tests under heat, moisture, and light exposure.

We weighed specified quantities of PO, PO-SECs, and Ca-Alg@PO-SECs powders and placed them into stability chambers under the following test conditions:

Heat Test: 60 °C, 60% humidity, samples sealed in clean containers.

Humidity Test: 25 °C, 90% humidity, samples left open in clean containers.

Light Stability Test: Light at 4500 ± 500 lx, controlled temperature.

All samples were stored for 10 days. Subsamples were collected on the 5th and 10th days.

Stability was quantitatively assessed by measuring mass variation, which reflects material loss or degradation under the tested conditions and serves as an indirect indicator of morphological and structural integrity.

### 2.8. In Vitro Release Kinetics

We examined the in vitro release kinetics of PO from Ca-Alg@PO-SECs composite microspheres. The tests were performed under simulated gastrointestinal conditions (SGF and SIF).

#### 2.8.1. Drug Release Behavior in Simulated Gastric Fluid (SGF)

We accurately weighed approximately 1.0 g of the prepared Ca-Alg@PO-SECs microspheres. These were dispersed in 10 mL of simulated gastric fluid (SGF). The release test was performed in a thermostatic shaking water bath at 37 ± 0.5 °C with a rotation speed of 100 rpm. At predetermined intervals (0.25, 0.5, 1, 2, 4, 6, 12, 24, 48, 60, and 72 h), we withdrew 1.0 mL of the release medium. Immediately after each withdrawal, we replaced this volume with an equal amount of preheated blank medium to maintain a constant volume. The collected samples were then centrifuged at 10,000 rpm for 20 min. The resulting supernatants were filtered through a 0.45 μm organic membrane filter before HPLC analysis under established chromatographic conditions.

The concentration of PO was determined using a standard calibration curve. All measurements were performed in triplicate.

The cumulative release percentage (Qₙ) was calculated using the following equation:

$$Q_{n}(\%)=[\sum (C_{i}\times V)+(C\times V)]/M_{\mathrm{total}}\times 100\%$$

$C_{i}$ and $C_{n}$ represent the PO concentrations (mg/mL) at the ith and nth sampling points, respectively; V is the sampling or total volume (mL); and $M_{\mathrm{total}}$ is the total amount of PO loaded in the sample (mg); n is the number of sampling points.

#### 2.8.2. Drug Release Behavior in Simulated Intestinal Fluid (SIF))

After SGF release, we used the remaining microspheres or a fresh sample for SIF release testing.

We accurately weighed approximately 1.0 g of Ca-Alg@PO-SECs. The sample was dispersed in 10 mL of SIF. The release test took place in a thermostatic water bath at 37 ± 0.5 °C with a shaking speed of −10,000 rpm.

At predetermined time points (0.25, 0.75, 1, 2, 4, 6, 12, 24, 48, and 60 h), we withdrew 1.0 mL of release medium, ensuring each sample was immediately replaced with an equal volume (1.0 mL) of isothermal blank medium to maintain the original volume. The collected samples were filtered through a 0.45 μm organic membrane to remove particulates before analysis. All samples were analyzed under the same chromatographic conditions described above. PO concentrations were determined using the calibration curve. Each sample was tested in triplicate to obtain mean values. The cumulative release rate (Qₙ) was calculated as described in Section 2.8.1.

### 2.9. Pharmacodynamic Evaluation of Acute Colitis

#### 2.9.1. Animal Experiment Design and Grouping

We obtained a total of 48 male SPF-grade C57BL/6J mice (8 weeks old, 22 ± 2 g) After 1 week of acclimatization, we randomly assigned the mice to 6 groups (*n* = 8 per group). We treated all mice by gavage once daily for seven days.

The groups were as follows: Control group (received sterile water and PBS by gavage). The other groups consisted of experimental and treatment groups, each receiving specified treatments by gavage according to the experimental design (full details of each group’s treatment regimen are provided in the accompanying table). Each treatment used 200 μL.

① Control group: received sterile water and PBS by gavage;
② Model group: received 3% DSS solution and PBS by gavage;
③ Positive drug group: received 3% DSS solution and mesalazine suspension (100 mg/kg/day);
④ PO group: received 3% DSS solution and Pogostemon oil suspension (20 mg/kg/day);
⑤ PO-SECs group: received 3% DSS solution and PO-loaded sporopollenin microcapsule suspension (equivalent to 20 mg/kg/day of PO);
⑥ Ca-Alg@PO-SECs group: received 3% DSS solution and Ca-Alg@PO-SECs suspension (equivalent to 20 mg/kg/day of PO).

On day 8, mice fasted for 24 h (with water). Blood was collected via retro-orbital puncture. Retro-orbital blood collection was performed under anesthesia by trained personnel, with strict control of sampling volume and frequency to minimize animal distress. Fasting was limited to a single 24 h period with free access to water and continuous monitoring. No unexpected mortality associated with these procedures was observed.

The mice were then euthanized by cervical dislocation. We removed the entire colon, measured its length.

#### 2.9.2. Physiological Parameter Monitoring

During the experiment, we monitored and recorded the body weight, food intake, and water consumption of mice in each group daily. This allowed us to assess the effects of DSS and different drug treatments on the mice’s basic physiological status and metabolism.

#### 2.9.3. Disease Activity Index (DAI) Score

The Disease Activity Index (DAI) is a quantitative measure of acute colitis severity. It reflects colonic injury; higher scores indicate greater severity. Each day, we assessed body weight loss, fecal consistency, and fecal blood. We calculated the DAI score, averaged from these three parameters, using the criteria in Table 1 and Formula.

#### 2.9.4. Serum Collection and Colon Length

Serum Collection: Whole blood from mice was collected via retro-orbital puncture into centrifuge tubes. Samples were allowed to clot at room temperature before centrifugation at 3000× *g* for 15 min at 4 °C. After centrifugation, the serum was divided into portions and stored at −80 °C for further analysis.

Organ Index: After serum collection, mice were euthanized by cervical dislocation. The heart, liver, spleen, lungs, and kidneys were dissected, blotted dry, and weighed to calculate organ indices.

Colon Tissue Processing. We carefully dissected the colon from cecum to rectum, measured its length, and photographed it. We divided the colon into three sections:

Histology: One section was fixed in 4% paraformaldehyde for subsequent paraffin embedding and histological examination.

Tissue Homogenate: Another section (100 mg of tissue) was homogenized in 9 volumes of pre-chilled PBS. We ground the tissue using a tissue homogenizer at 4 °C and centrifuged it at 13,000× *g* for 10 min. The supernatant was stored at −80 °C for biochemical analysis.

Molecular Analysis: The remaining portion was wrapped in aluminum foil, rapidly frozen in liquid nitrogen, and stored at −80 °C for molecular biology analysis.

#### 2.9.5. ELISA Assay

We measured the levels of L-6, IL-1β, and IL-10 in the serum and colon tissues of mice using ELISA kits (Hangzhou HuaAn Biotechnology Co., Ltd., Hangzhou, China). The kits were allowed to equilibrate at room temperature for 60 min before use. Following the manufacturer’s instructions, we added standard samples and test samples to pre-coated 96-well plates. After incubation, washing, addition of biotinylated detection antibodies, streptavidin-HRP conjugate, and substrate solution, we measured absorbance at 450 nm using a CLARIOSTAR microplate reader (BMG LABtech, Ortenberg, Germany). We calculated cytokine concentrations in the samples using the standard curve generated from the standard samples.

## 3. Results and Discussion

### 3.1. Effect of Acid Hydrolysis Time on the Structure of Pollen-Spore Encapsulation and Protein Removal Rate

This study examines how acid hydrolysis time influences particle size and protein removal in pollen from Tea, rose, sunflower, and rapeseed. The aim is to determine optimal conditions for producing SECs, which are hollow shells left when acid hydrolysis removes the cell’s nucleus, cytoplasm, and proteins, leaving only the outer exine wall.

#### 3.1.1. Particle Size Change in Raw and Acid-Hydrolyzed Pollen

**Figure 1:**
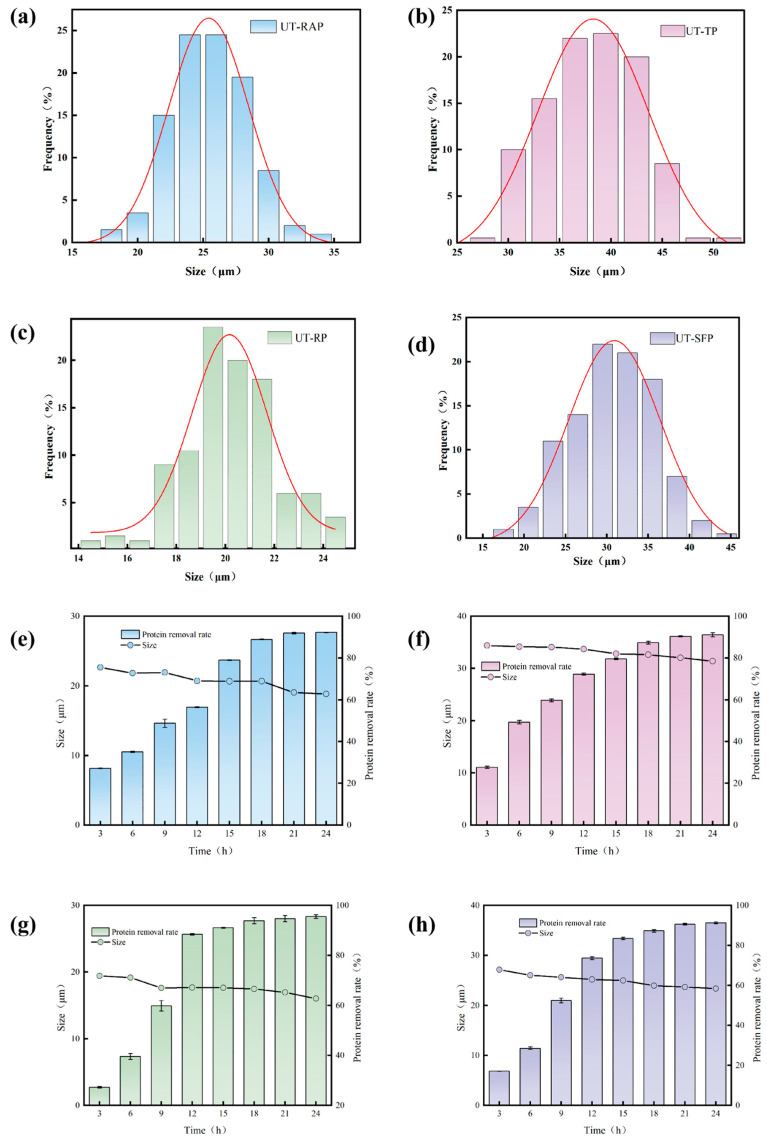
Characterization of particle size and protein removal in four pollen species during acid–base treatment. (**a**–**d**) Original particle sizes of rapeseed, tea, rose and sunflower pollen, respectively. (**e**–**h**) Time-dependent variations in protein removal rate and particle size of rapeseed, tea, rose, and sunflower pollen during the acid–base treatment process. The bars correspond to the protein removal rate (%), while the line plots represent the average particle size (μm). Data are presented as mean ± SD (n = 3), with error bars representing the standard deviation (SD).

Figure 1a–d shows sharp differences in particle sizes among the four pollens: rapeseed pollen is 25.42 ± 0.11 μm, tea pollen is 38.27 ± 0.31 μm, rose pollen is 20.17 ± 0.16 μm, and sunflower pollen is 30.90 ± 0.31 μm. Acid hydrolysis led to an observable reduction in particle size across all four pollen types and resulted in the removal of the outer exine structure, as evidenced by microscopic characterization. Rapeseed pollen showed the greatest size reduction (about 25.81%). This may be because it has a thinner outer wall or a higher protein-to-lipid ratio. These factors can cause the sporopollenin shells to contract further after the inner parts are removed.

#### 3.1.2. Kinetic Effect of Acid Hydrolysis Time on Protein Removal Rate

Figure 1e–h shows how protein removal in pollen changes over time during acid hydrolysis, breaking the process into two distinct stages:

Rapid Removal Stage (3 to 18 h): During this phase, acid rapidly degrades readily accessible proteins found on the particle’s surface and outer wall, resulting in a high level of protein removal. Plateau and Slow Stage (18 to 24 h): At this stage, acid removes almost all accessible proteins, raising the removal rate above 95%. Extending the time yields little additional removal.

#### 3.1.3. Kinetic Effect of Acid Hydrolysis Time on Particle Size

Figure 1e–h shows that as acid hydrolysis time increases, the particle size gradually decreases without drastic fluctuations in distribution. This trend indicates that, despite ongoing protein removal, the SECs main chain structure remains intact and chemically resistant during acid hydrolysis.

The removal of endogenous proteins and lipids reduces particle size by causing the SECs to lose internal support and contract. This steady change demonstrates the SECs’s high acid tolerance and ensures the structural stability of the final product by removing only surface materials.

### 3.2. Morphological Analysis and Structural Stability of Spore Encapsulated Microspheres

This section analyzes the natural shapes (morphology) of different pollen types. It also explores the structural changes in sunflower pollen during preparation. The effect of acid hydrolysis duration on the microporous structure of SECs was assessed using scanning electron microscopy (SEM).

#### 3.2.1. Original Morphology and Structural Stability

**Figure 2:**
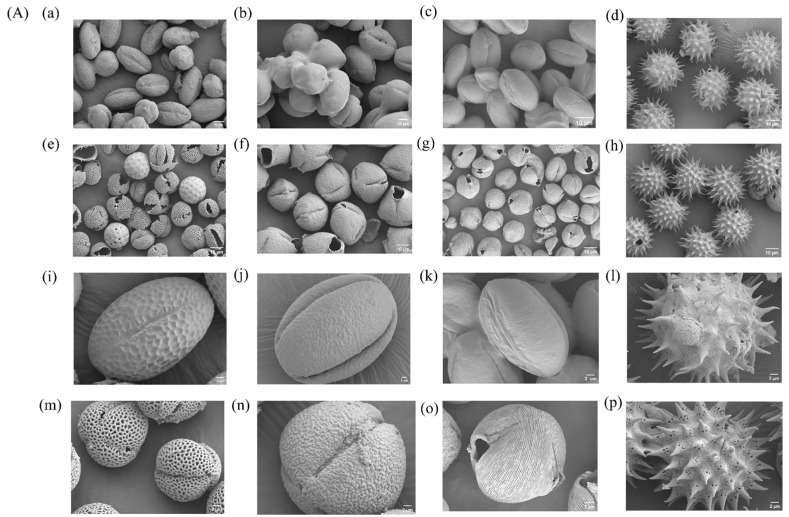

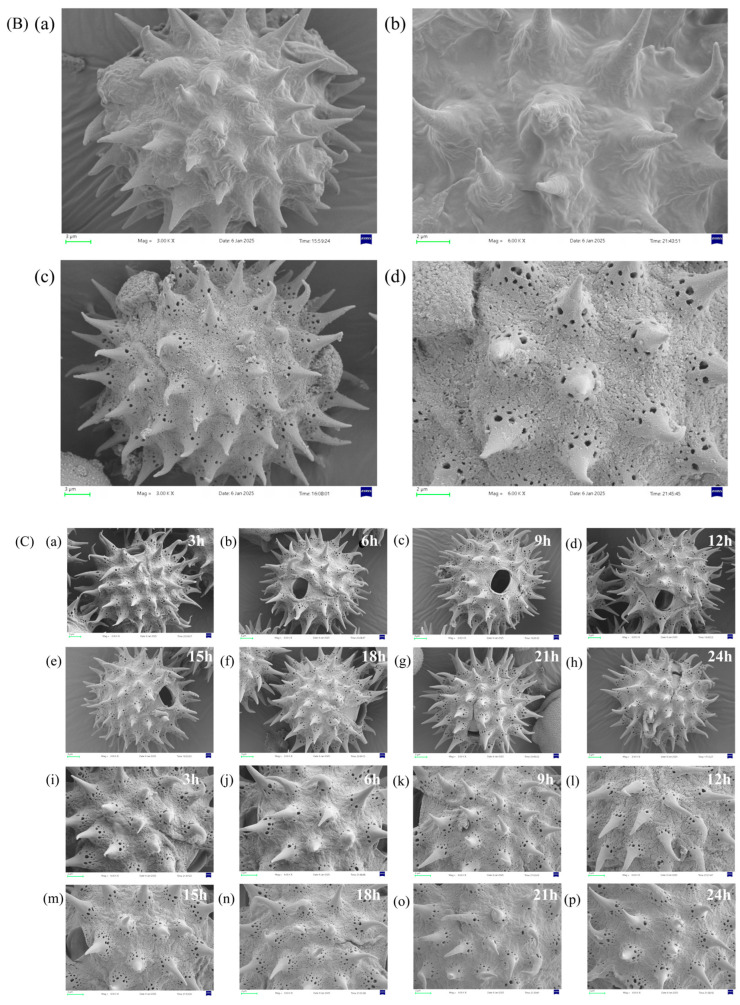
Morphological evolution of four pollen types before and after acid hydrolysis. (**A**) SEM images of rapeseed, tea, rose and sunflower pollen. Panels (**a**–**d**) show the native pollen grains, and panels (**e**–**h**) show the corresponding SECs after acid hydrolysis (scale bars = 10 μm). Panels (**i**–**l**) display the surface microstructures of the native pollen, while panels (**m**–**p**) present the surface morphology of the SECs from all four species at higher magnification (scale bars = 2 μm). (**B**) Magnified SEM images of SFP before and after treatment. Panel (**a**) shows UT-SFP (scale bar = 3 μm), and panel (**b**) shows UT-SFP (scale bar = 2 μm). Panel (**c**) shows AT-SFPS (scale bar = 3 μm), and panel (**d**) illustrates AT-SFPS at higher magnification (scale bar = 2 μm); (**C**) Time-dependent morphological changes in sunflower pollen during acid hydrolysis. Panels (**a**–**h**) show the structural transition at lower magnification (scale bars = 3 μm), and panels (**i**–**p**) show the detailed surface (scale bars = 2 μm).

As shown in Figure 2A, the four types of raw pollen each display unique morphologies, consistent with previous literature. After phosphoric acid treatment, the resulting SECs retained an overall morphology similar to that of untreated pollen, indicating the preservation of a chemically stable and mechanically robust sporopollenin framework. Notably, they retain their original shape and size even after exposure to strong acids.

#### 3.2.2. Morphological Changes During SECs Preparation

Figure 2B illustrates morphological changes in sunflower pollen during preparation. Initially, the raw pollen surface is relatively smooth and coated with Pollen Kitt—a protective, sticky substance (lipids, proteins, and pigments) that acts as a physical barrier.

Subsequent chemical treatments further alter the structure. Specifically, phosphoric acid treatment and acid hydrolysis completely remove the Pollen Kitt layer as well as internal proteins and lipids. This purifies the pollen, exposing its rough, porous structure and transforming the originally solid structure into a hollow, porous cavity.

This morphological transformation provides the structural basis for the SECs’ adsorption properties:

Surface roughening increases the specific surface area (total surface area per unit mass) and thereby boosts drug adsorption onto SECs.

The porous network and hollow structure allow oily substances to quickly penetrate the cavity via capillary action (the movement of liquids into small spaces due to surface tension). This design ensures efficient filling and storage.

Surface adsorption and cavity filling together greatly enhance the SECs’ ability to extract oily drugs from the aqueous phase.

#### 3.2.3. Effect of Acid Hydrolysis Time on the Microporous Structure

Shifting focus to structural effects, Figure 2C shows that varying acid hydrolysis times profoundly shape the microporous structure on the SEC surfaces.

At the onset of acid hydrolysis, micropores rapidly form on the outer wall. Extending the hydrolysis time removes deeper layers and creates larger pores.

The result is a more distinct honeycomb-like within the exine wall, which not only increases the chemical contact area but also significantly improves channel formation for capillary-driven adsorption.

However, it should be noted that excessively prolonged acid hydrolysis may also introduce potential trade-offs. In pollen images obtained after prolonged acid hydrolysis (Figure S1), structural rupture of pollen particles was clearly observed. Therefore, an 18 h hydrolysis time was selected as a balanced condition that sufficiently develops the accessible porosity while maintaining overall structural integrity of the SECs.

### 3.3. Fourier Transform Infrared (FTIR) Analysis

**Figure 3:**
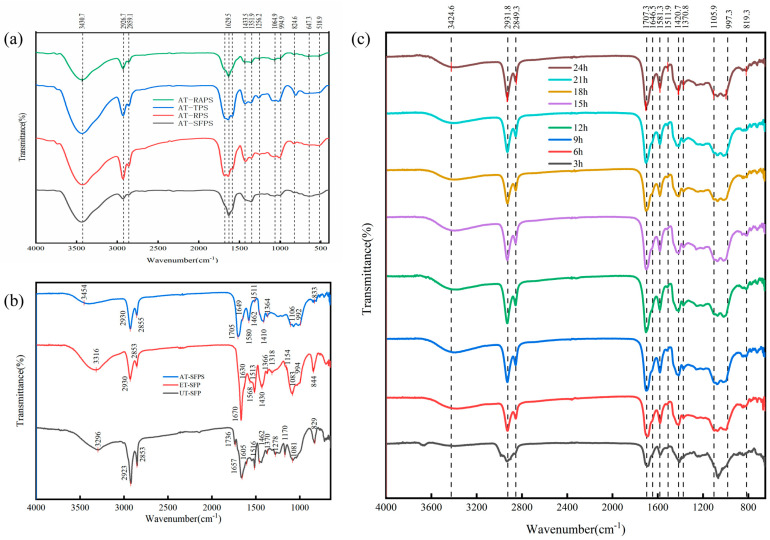
Fourier-transform infrared (FTIR) spectra of pollen-derived samples. (**a**) FTIR spectra of rapeseed, tea, rose, and sunflower pollen. (**b**) FTIR spectra of sunflower pollen before treatment, after ethanol washing, and after phosphoric acid hydrolysis. (**c**) FTIR spectra of sunflower pollen-derived residues obtained after phosphoric acid hydrolysis for different durations.

This section uses Fourier Transform Infrared (FTIR) spectroscopy to evaluate the chemical composition and purification effectiveness of SECs. It also assesses changes in functional groups during their preparation. To facilitate comparison, the FTIR spectra in Figure 3a,c were presented with an emphasis on representative characteristic peaks, highlighting the structural similarity of sporopollenin across different sources and processing conditions.

#### 3.3.1. Chemical Similarity of Spores from Different Sources

Figure 3a shows the FTIR spectra of four acid-hydrolyzed SECs samples (AT-RAPS, AT-TPS, AT-RPS, AT-SFPS).

The four SECs share consistent peak positions in the characteristic absorption region (3600–1300 cm^−1^), indicating similar chemical composition and molecular structure. Differences in the fingerprint region (1300–400 cm^−1^) reflect slight microstructural variations.

A broad absorption peak around 3430 cm^−1^ indicates O-H/N-H stretching vibrations, suggesting the presence of hydroxyl or amino groups. AT-TPS and AT-RAPS show stronger absorption, indicating higher levels of polar groups or residual proteins.

Peaks at 2926 cm^−1^ and 2859 cm^−1^ correspond to aliphatic C-H stretching vibrations, confirming the presence of stable hydrocarbon chains in the SECs. Peaks at 1256, 1065, and 995 cm^−1^ are due to C-O stretching vibrations, originating from polysaccharides, esters, or ether bonds. Characteristic peaks in the fingerprint region (<900 cm^−1^) at 824, 647, and 519 cm^−1^ relate to the inherent aromatic skeletal vibrations of the SECs, indicating good structural integrity of the outer shell.

In summary, all four SECs samples share similar types of functional groups and peak positions. However, differences in the relative spectral contributions of polar groups, lipid residues, and polysaccharide/ester-related bands were observed among the four SEC samples, reflecting variations in their purification outcomes.

#### 3.3.2. Purification Effect of SECs

Figure 3b presents the FTIR spectra of sunflower SECs at different purification stages.

The absorption peak at 1700 cm^−1^ is typically associated with C=O stretching vibrations (e.g., esters, aldehydes, or proteins). This peak is prominent in the untreated sample (UT-SFP) but is attenuated or absent in the final product (AT-SFPS), in agreement with the expected removal of protein- and ester-related components during acid hydrolysis. This reduction directly shows the efficiency of protein and ester removal during acid hydrolysis.

After acid hydrolysis, peaks at 2926 cm^−1^ and 2850 cm^−1^ show typical aliphatic C–H stretching vibrations, primarily from lipid components. The decreased intensity of these peaks indicates the removal of most soluble lipids, while the hydrocarbon chain structure of the SECs encapsulation was retained.

Peaks at 1250 cm^−1^ and 1050 cm^−1^, attributed to C-O stretching vibrations, suggest a high enrichment of polysaccharides, alcohols, or esters in the final sample.

FTIR analysis demonstrates that acid hydrolysis effectively removes proteins and soluble lipids from the pollen while preserving the core chemical skeleton of the SECs encapsulation.

#### 3.3.3. Effect of Acidolysis Time on Functional Group Changes

Figure 3c shows that the FTIR spectra of pollen shell samples treated for various acidolysis times (3 h to 24 h) exhibit similar peak patterns, but the intensities of the characteristic absorption peaks systematically shift with the reaction time, reflecting the dynamics of the purification process.

The absorption peak for O–H stretching vibration at 3434 cm^−1^ gradually weakened with increasing acidolysis time, indicating the removal of surface water and hydrogen-bonded water.

The C=O stretching vibration peak at 1707 cm^−1^ significantly decreases between 3 and 12 h, indicating that acidolysis effectively removes protein and other carbonyl-containing components. After 18 h, this peak’s intensity plateaus, showing that the removable components have largely been depleted.

The absorption peaks for aliphatic C-H stretching vibrations (2923 cm^−1^ and 2850 cm^−1^) and the aromatic C=C stretching vibration peak (1600 cm^−1^) showed little variation.

These signals indicate that the aromatic backbone and aliphatic hydrocarbon chains of the pollen shells remain intact during acidolysis. Acidolysis primarily acts on the outer layers, gradually penetrates deeper layers, and effectively removes proteins, esters, and polar groups. The purification process stabilizes around 18 h.

### 3.4. Study of the Dispersibility, Affinity, and Surface Wettability of Pollen Spore Shells

With the molecular structure confirmed, the next step is to evaluate the essential properties of SECs. These include dispersibility, affinity, and surface wettability. This assessment helps determine their potential for practical use.

#### 3.4.1. Dispersibility and Affinity of SECs in Different Solvents

**Figure 4:**
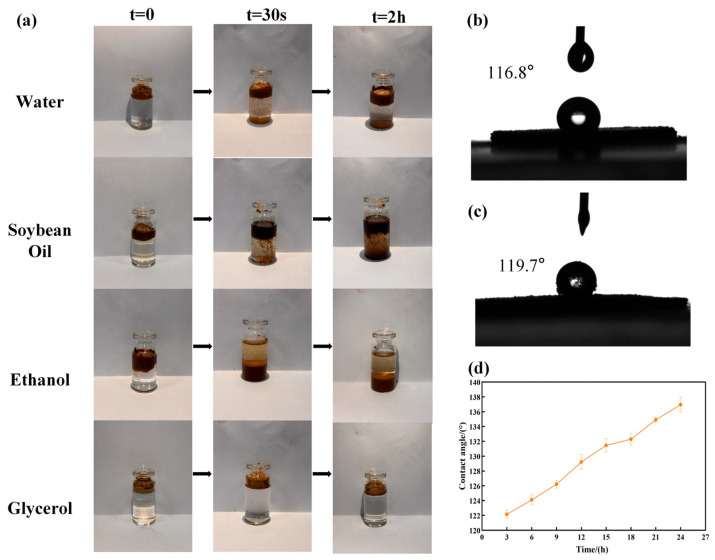
Dispersibility, solvent affinity, and surface wettability of sunflower pollen and SECs. (**a**) Dispersibility and solvent affinity of sunflower pollen in different liquids. (**b**) Water contact angle of raw sunflower pollen. (**c**) Water contact angle of defatted sunflower pollen. (**d**) Water contact angles of sunflower pollenin after acid treatment for different durations, Data are mean ± SD (n = 3). Statistical significance determined by unpaired two-tailed *t*-test, *p* < 0.05 vs. raw pollen).

Figure 4a illustrates the dispersion and interfacial behavior of SECs in four different polarity systems (water, soybean oil, ethanol, and glycerol), qualitatively reflecting their surface affinity:

Water (high polarity): SECs remained largely at the liquid–air or liquid–water interface. Partial immersion was observed even after 2 h. This indicates weak interactions with water molecules, poor dispersibility, and slow interfacial adsorption, consistent with their hydrophobic nature (Video S1).

Soybean oil (non-polar): SECs rapidly wetted the oil phase and accumulated at the oil–water interface. Partial interfacial immobilization occurred within minutes. This reflects oleophilicity and a strong affinity for non-polar media (Video S2, Figure S3).

Ethanol (polar organic solvent): SECs quickly aggregated and localized at interfaces within 30 s. Most particles remained interface-associated, rather than fully settling at the bottom. This behavior likely arises from reduced electrostatic repulsion in ethanol, which promotes interfacial adsorption and aggregation.

Glycerol (high viscosity, moderate polarity): SECs primarily stayed near the liquid surface. They showed limited partial immersion and interface-associated retention at both 30 s and 2 h. This suggests intermediate interaction strength between SECs and glycerol, consistent with their polarity-dependent affinity.

Overall, these observations indicate that SEC dispersibility and localization are governed by their superhydrophobic and oleophilic surface properties. Particles are preferentially retained at liquid interfaces rather than undergoing classical sedimentation.

#### 3.4.2. Contact Angle Analysis and Superhydrophobic Performance

To quantitatively assess the surface wettability of SECs, we studied their water contact angle (θ). To quantitatively assess the surface wettability of SECs, we studied their water contact angle (θ). The water contact angle of untreated sunflower pollen was 116.8° ± 0.6° (Figure 4b), classifying it as a hydrophobic material. After ethanol treatment, the water contact angle of the sunflower spore shells increased to 119.7° ± 0.5° (Figure 4c), representing a statistically enhancement in surface hydrophobicity compared with untreated pollen (Figure S2, *p* < 0.001).

As the acidolysis time increased, the contact angle gradually increased, indicating a decline in hydrophilicity and an enhancement of oleophilicity. Specifically, after 18 h of acidolysis, the contact angle of the SECs exceeded 130° (Figure 4d). This change is consistent with the FTIR results in Section 3.3.3: Longer acidolysis times led to a decrease in hydrophilic groups (such as O–H/N–H) and protein residues on the pollen shell surface, exposing more hydrophobic spore shell structures (hydrocarbon chains and aromatic rings), thus enhancing the hydrophobicity of the material.

Combined with the SEM analysis in Section 3.2, this superhydrophobic performance is the result of the synergistic effect between the inherent hydrophobic chemical properties of SECs and their rough, porous structure.

### 3.5. Oil Adsorption Performance Study

#### 3.5.1. Adsorption Capacity of SECs from Different Sources

**Figure 5:**
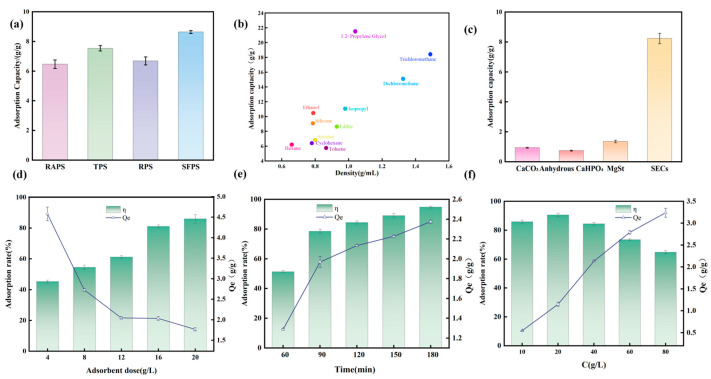
Adsorption performance of pollen SECs toward oils. Data are presented as mean ± SD (n = 3). (**a**) Comparison of the adsorption capacities of four pollen spore proteins toward soybean oil; (**b**) Adsorption capacity of SFPS in various organic solvents; (**c**) Comparative evaluation of the adsorption performance of SFPS and other conventional oil adsorbents; (**d**–**f**) Influence of experimental parameters on the adsorption performance of SFPS. The bars correspond to the adsorption rate (η, %), while the line plots represent the adsorption capacity (Q_e_, g/g). (**d**) Correlation between the dosage of sunflower sporopollenin and its adsorption capacity and efficiency; (**e**) Effect of adsorption time on adsorption capacity and efficiency; (**f**) Influence of the initial soybean oil concentration on adsorption capacity and efficiency.

Oil-absorption ability is a key trait of oleophilic and hydrophobic materials. We examined this for SECs from four plant pollens. Although their oleophilic characteristics were similar on a large scale, oil adsorption performance varied (Figure 5a). SFPS had the highest capacity (8.64 g/g). RAPS had the lowest. This variation is likely due to differences in particle size, surface roughness, or remaining polar groups. These data are important for comparing the inherent efficiency of SECs.

Given SFPS’s oil-loading capacity and structural robustness, SFPS were selected as the optimal carrier for all subsequent encapsulation and pharmacodynamic investigations.

#### 3.5.2. Adsorption Capacity of Sunflower SECs for Different Solvents

To assess the potential of AT-SFPS as a broad-spectrum oil carrier, its adsorption behavior toward liquids with varying polarity and density was examined using a comparative oil retention assay. Figure 5b shows that solvent properties shape the results.

The findings reinforce that AT-SFPS exhibits superhydrophobic and strong oleophilic properties, with adsorption driven not only by the SECs’ non-polar, lipophilic nature, in accordance with the ‘like dissolves like’ principle, but also by powerful physical mechanisms inherent to its architecture. Specifically, while chemical affinity dictates the selective wetting of the SEC surface, it works in synergy with strong capillary action provided by the unique hollow-shell structure. The micro-scale lumen of the sunflower SECs facilitates rapid liquid wicking, where capillary pressure effectively ‘pumps’ and traps non-polar molecules within the internal cavity, leading to the exceptionally high Q_e_ (27.64 g/g). Furthermore, the hierarchical spiked morphology of the exine surface complements this process by enhancing surface adsorption through increased contact area and van der Waals interactions. In short, the superior performance of AT-SFPS stems from the combined effects of its chemical lipophilicity and its naturally engineered physical suction capabilities.

#### 3.5.3. Comparison of Adsorption Capacity for Soybean Oil by Different Adsorbents

We further compared oil adsorption by testing AT-SFPS and common absorbents (calcium carbonate, anhydrous dicalcium phosphate) using an oil-leakage method. Equal soybean oil were mixed with each adsorbent. After absorption and compaction, we checked for oil leakage. This method provides a direct comparison of efficiency.

The results in Figure 5c show that AT-SFPS (8.19 g/g) adsorbs significantly more oil than conventional excipients. For the same oil amount, less AT-SFPS is required than with standard excipients, indicating its efficient oil-loading capacity. This performance is linked to the hollow structure of SECs, which aids oil uptake by internal cavities and surface adsorption. These properties suggest AT-SFPS is promising for large-scale pharmaceutical oil formulations.

#### 3.5.4. Adsorption Kinetics and Parameter Optimization

This study systematically assessed the impact of adsorbent dosage, adsorption time, and initial soybean oil concentration on the adsorption capacity (Q_e_) and efficiency (η) of SECs for oily substances. These results provide a robust basis for optimization.

(1) Effect of Adsorbent Dosage.

To investigate the effect of SEC dosage on adsorption capacity and efficiency for soybean oil, the following observations were made (Figure 5d).

Low Dosage Range (4–12 g/L): In this range, the adsorption efficiency (η) increased from 44.96 to 61.22%, showing a minor increase of 16.26% for each additional gram of SECs. Conversely, the adsorption capacity (Q_e_) decreased from 4.58 g/g to 2.05 g/g.

Interpretation: When the SEC dosage is low, more target substance molecules can be contacted per unit mass of SECs, resulting in a higher Q_e_. However, the overall removal efficiency (η) is lower due to the insufficient total number of available SECs.

Intermediate Dosage Range (12–20 g/L): As the SEC dosage increased across this range, the adsorption efficiency (η) significantly increased from 61.22% to 86.01%. However, the adsorption capacity (Q_e_) continued to decrease from 2.05 g/g to 1.77 g/g.

Interpretation: Increasing the adsorbent dosage provides more total adsorption sites, thereby significantly improving overall removal efficiency (η). However, at higher concentrations, decreased contact probability between SECs and target substances results in underutilized adsorption sites, thereby reducing Q_e_.

When the dosage reached 16 g/L, the adsorption efficiency exceeded 81%. Increasing the dosage further to 20 g/L increased efficiency to 86.01%, but Q_e_ decreased further to 1.77 g/g.

In summary, based on these results,16 g/L was selected as the optimal dosage. This concentration balances high adsorption efficiency and avoids excessive use of adsorbent material, providing a foundation for examining the effects of additional parameters.

(2) Effect of Adsorption Time (Adsorption Kinetics).

Building on the previously identified optimal dosage, the effect of adsorption time on soybean oil capacity and efficiency was examined (Figure 5e).

Rapid Adsorption Phase (60–90 min):The adsorption efficiency (η) and Q_e_ increased rapidly. Efficiency rose from 51.35% to 81.14% and Q_e_ increased from 1.29 g/g to 2.03 g/g.

The initial phase is characterized by a rapid increase in adsorption efficiency, attributed to the availability of many active sites on the SEC surface. Oil molecules diffuse quickly and bond during this stage, indicating that adsorption is mainly controlled by external mass transfer.

Slow Adsorption Phase (90–150 min):In the subsequent phase, the rate of increase in efficiency slowed significantly and eventually stabilized. The time required increased from 90 min to 180 min, Beyond 150–180 min, efficiency showed almost no further change. Q_e_ increased slowly, from 2.03 g/g to 2.17 g/g.

This second phase marks the point at which most surface-active sites were occupied. The process slowed mainly due to the diffusion of soybean oil into SECs’ internal cavities (intraparticle diffusion), thereby significantly slowing overall adsorption.

Consequently, given the limited increase in efficiency beyond 90 min, 90 min was designated as the optimal adsorption time, and this criterion was used in subsequent steps.

(3) Effect of Initial Soybean Oil Concentration.

With optimized dosage and time parameters established, the effect of initial soybean oil concentration on adsorption capacity and efficiency was evaluated (Figure 5f).

Low Concentration Range (10–20 g/L—Insufficient Oil Phase)

As the soybean oil concentration increased, the adsorption efficiency (η) rose rapidly from 85.81% to 90.54%, and the adsorption capacity (Q_e_) increased from 0.55 g/g to 1.1 g/g.

The SECs’ adsorption sites were abundant, allowing increased contact with soybean oil as its concentration rose. This competition-free environment led to higher efficiency and an increased Q_e_.

High Concentration Range (20–80 g/L):As the oil concentration increased across the high range, the removal efficiency (η) fell from 90.54% to 64.78%. With the number of SEC sites fixed, the high oil concentration resulted in fewer sites available per oil molecule, thereby decreasing efficiency.

As the soybean oil concentration increased from10 g/L to 80 g/L Q_e_ gradually increased from 0.55 g/g to 3.24 g/g. In the range of 10–20 g/L, Qe increased rapidly. However, in the range of 20–80 g/L, the rate of Q_e_ increase slowed down significantly. This indicates that SECs’ adsorption sites were gradually nearing saturation.

Therefore, 20 g/L was selected as the optimal concentration for subsequent experiments, as it yields the highest Q_e_ before efficiency declines, aligning with previously determined conditions.

### 3.6. Adsorption Performance of SECs for Pogostemon Oil (PO-SECs) and Optimization of Parameters

This section investigates the adsorption performance of SECs using PO as a model oily drug. Three experimental method-passive adsorption, vacuum adsorption, and selective adsorption-are employed to systematically examine the impact of SECs dosage, adsorption time, and PO concentration on adsorption efficiency (η) and adsorption capacity (M).

#### 3.6.1. Consistent Patterns of Adsorption Parameter Effects

**Figure 6:**
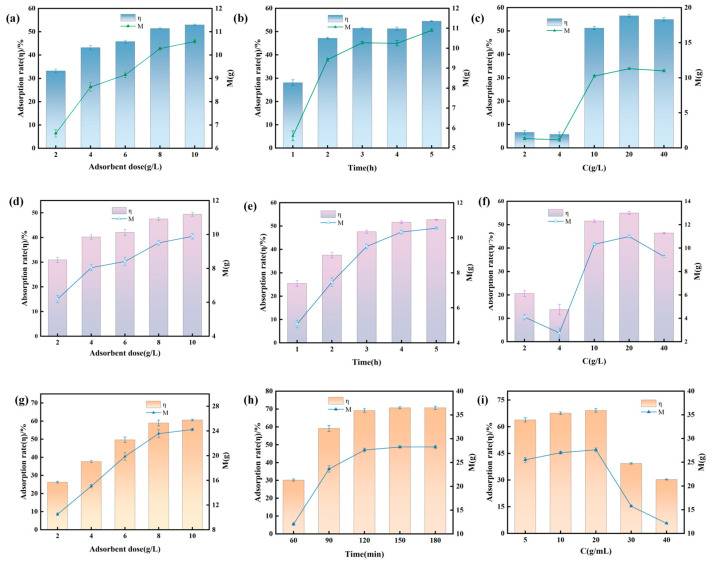
Effects of different adsorption conditions on the adsorption capacity and efficiency of SECs. The bars correspond to the adsorption rate (η, %), while the line plots represent the adsorption capacity (M, g/L). Data are presented as mean ± SD (*n* = 3). Vacuum adsorption: (**a**) Influence of SEC dosage. (**b**) Influence of adsorption time. (**c**) Influence of soybean oil concentration. Passive adsorption: (**d**) Influence of SEC dosage. (**e**) Influence of adsorption time. (**f**) Influence of soybean oil concentration. Selective adsorption: (**g**) Influence of SEC dosage. (**h**) Influence of adsorption time. (**i**) Influence of soybean oil concentration.

In Figure 6, all three adsorption methods, the effects of SECs dosage, adsorption time, and PO concentration on adsorption performance show consistent patterns:

(1) Effect of Adsorbent Dosage.

η increases with SECs dosage because more adsorption sites capture target substances. M gradually slows as the total amount of the target substance is fixed and the unit adsorbent loading capacity decreases. Eventually, the system approaches adsorption saturation.

(2) Effect of Adsorption Time (Kinetics).

Initial Stage (Rapid Adsorption): In the early stage (e.g., passive adsorption 1–3 h, vacuum adsorption, 1–2 h, selective adsorption 1–4 h), efficiency and adsorption capacity increase rapidly. This indicates that the adsorbent is fully contacting the oil and that the active sites are quickly occupied.

Later Stage (Stabilisation): In the later stage, the growth rate slows and stabilizes (approaching equilibrium), indicating that the adsorption sites are nearly saturated and that the target substance must overcome greater internal diffusion resistance to continue binding.

(3) Effect of Pogostemon Oil Concentration.

Low Concentration Stage: In the low concentration stage of PO (e.g., passive adsorption 2–20 g/L, vacuum adsorption 2–20 g/L, selective adsorption 10–40 g/L), both efficiency and adsorption capacity increase with concentration. This results from a higher probability of contact between oil molecules and adsorption sites.

High Concentration Stage: After the concentration exceeds a threshold, both efficiency and adsorption capacity decrease. This shows that the total number of oil molecules far exceeds the total number of adsorption sites, leading to more unadsorbed oil molecules and a lower removal rate (η).

#### 3.6.2. Comparison of the Three Adsorption Methods and Selection of Optimal Parameters

Due to differences in experimental conditions (e.g., environmental pressure, solvent selection), the optimal parameters for the three adsorption methods vary, as summarised in Table 2.

Among all the tested adsorption methods, selective adsorption demonstrated significant advantages. SECs showed a much higher adsorption capacity (M) for Pogostemon oil (27.64 g/g) compared to passive adsorption (11.29 g/g) and vacuum adsorption (10.99 g/g), with a higher efficiency limit (69.11%). This strongly confirms the powerful adsorption capacity and selectivity of SECs for volatile oils (Video S3), particularly due to the solvent-assisted mechanism of selective adsorption.

This section clarifies the optimal parameter settings for different experimental conditions. It provides crucial insights into the practical application of SECs as an oil drug absorbent, especially in developing formulations for Traditional Chinese Medicine essential oils.

### 3.7. Characterization of Microcapsules

#### 3.7.1. Microcapsule Appearance

**Figure 7:**
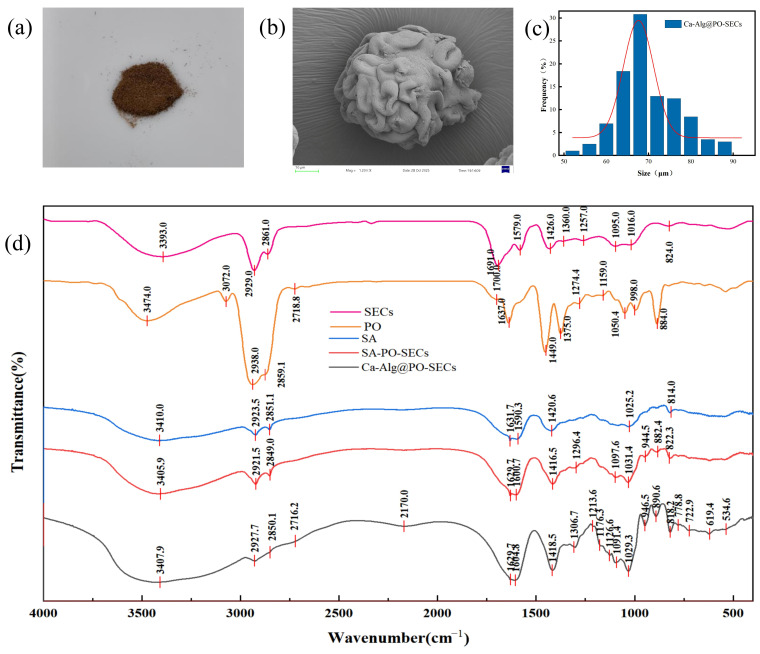
Characterization of Ca-Alg@PO-SECs composite microspheres. (**a**) Photograph of the Ca-Alg@PO-SECs powder. (**b**) SEM image of the Ca-Alg@PO-SECs composite microspheres (scale bars = 10 μm). (**c**) Particle size distribution of the Ca-Alg@PO-SECs composite microspheres. (**d**) FTIR spectra of sphingosine, patchouli oil, sodium alginate, PO-SECs, and Ca-Alg@PO-SECs composite microspheres.

Figure 7a shows the obtained microcapsule powder exhibited a brown appearance with satisfactory uniformity.

#### 3.7.2. Ca-Alg@PO-SECs Composite Structure Morphology

The composite structure is roughly spherical and has a highly irregular surface. Particle size analysis showed an average diameter of 67.60 ± 0.61 μm (Figure 7b).

The main feature is a pronounced, pleated (convoluted) morphology. This morphology (Figure 7c) results from rapid water loss from the hydrogel (Ca-Alg) shell during vacuum drying, causing the shell to contract and fold inward, tightly enveloping the core. Importantly, despite this transformation, the overall macroscopic shape retains the contour of the original SECs core.

Building on these observations, the Ca-Alg shell stays tightly around the core without rupturing during shrinkage. The Ca-Alg coating remains mechanically tough and highly crosslinked, maintaining the composite structure despite desiccation-induced stress. This confirms successful encapsulation of the oil-loaded core (PO-SECs).

Finally, this image confirms the formation of the Ca-Alg coating on the oil-containing SECs, providing a reliable, robust composite structure for subsequent in vitro release and stability testing.

#### 3.7.3. Stability Experiments

The stability of free PO, PO-SECs, and Ca-Alg@PO-SECs was evaluated under extreme temperature, humidity, and light using percentage weight mass (%). The results demonstrate that the dual-layer Ca-Alg@PO-SECs system provides enhanced resistance to volatilization and environmental stress under the tested conditions (Table 3).

At high temperatures, free PO underwent rapid and extensive weight loss, whereas encapsulation mitigated this loss. PO-SECs had a greatly reduced volatilization rate, confirming their role as an initial adsorption barrier. Ca-Alg@PO-SECs exhibited minimal weight change under high-temperature conditions, suggesting that the Ca-Alg layer acts as an effective physical barrier against thermal volatilization.

Under high humidity, free PO degradation (oxidation and volatilization) accelerated compared to normal conditions, while encapsulation offered distinct protection mechanisms: PO-SECs had reduced volatilization, indicating a modest initial adsorption barrier. Ca-Alg@PO-SECs showed a slight weight increase due to the hygroscopic Ca-Alg layer. Hydration of carboxyl groups and calcium-ion crosslinking contributed. More significantly, hydrated calcium alginate (Ca-Alg) impedes the diffusion of oxidative species (such as O_2_) to the carrier core, substantially reducing hydro-induced oxidative degradation. No oil loss or rupture confirmed stability against humidity-driven degradation.

Strong light exposure typically increases PO oxidation and volatilization. SECs provided measurable protection, reducing PO’s light-induced oxidation. Ca-Alg@PO-SECs exhibited exceptional light stability with almost no weight loss. The opaque calcium alginate (Ca-Alg) layer acts as an effective barrier, greatly reducing light-triggered damage to PO from ROS and helping to keep the compound’s natural antioxidant strength.

These results (Figure S4) demonstrate that PO-SECs improve thermal, humidity, and light stability compared to free PO, and Ca-Alg@PO-SECs exhibit environmental tolerance and essential oil retention. The hierarchical barrier architecture, comprising SECs and Ca-Alg, minimizes volatile losses while significantly impeding the ingress of environmental stressors, such as light, oxygen, and moisture. This protection preserves PO’s intrinsic antioxidant capacity, providing a robust material basis for subsequent drug-delivery and sustained-release applications.

#### 3.7.4. FTIR Analysis

Fourier Transform Infrared (FTIR) spectroscopy was used to detect the characteristic peaks of the drug in blank excipients, confirming whether the drug was successfully encapsulated into the excipients.

As shown in Figure 7d, SECs exhibits a distinct -OH stretching vibration peak at 3393.0 cm^−1^, attributed to the phenolic hydroxyl groups on its surface and the hydroxyl groups in residual polysaccharides; at 2927 cm^−1^ and 2854 cm^−1^, it shows aliphatic C–H stretching vibrations. The absorption peak at 1691.0 cm^−1^ corresponds to C=O stretching vibrations, characteristic of ester or carboxylic acid groups in SECs.

PO shows an -OH stretching vibration at 3474.0 cm^−1^. At 2938.0 cm^−1^ and 2859.1 cm^−1^, it presents characteristic aliphatic C-H stretching double peaks, typical of fatty chains, while the C=O peak at 1637 cm^−1^ corresponds to ester or aldehyde groups. The C–H bending vibrations occur at 1449 cm^−1^ and 1375 cm^−1^, while C-O stretching at 1159 cm^−1^ and 998.0 cm^−1^ indicates alcohol or ester functionalities. Additionally, a peak at 884.0 cm^−1^ represents the C-H out-of-plane bending vibration of a terpene skeleton (such as trisubstituted olefins). This spectrum reflects the typical structure of terpenes and alcohol-esters.

Sodium alginate (SA) exhibits a broad -OH absorption band at 3410.0 cm^−1^, with characteristic peaks at 1631.7 cm^−1^ and 1420.6 cm^−1^ corresponding to carboxylate (-COO-) groups, exhibiting both asymmetric and symmetric stretching vibrations. Alginate is a polysaccharide containing abundant carboxylate groups. The peak at 1025.2 cm^−1^ corresponds to C-O stretching, indicating the characteristic structure of sodium alginate with carboxylate and polysaccharide backbones.

SA-PO-SECs are in a physically mixed state, with the -OH peak widening significantly, likely due to overlapping peaks from PO and SECs. The C=O peak at 1629.7 cm^−1^ weakens, suggesting that PO molecules partially interact with active sites on the carrier surface. This indicates that PO has been adsorbed into the SA matrix, undergoing hydrogen bonding or hydrophobic interactions. The spectrum shows a peak overlap between SA and PO-SECs without significant red-shifting or new peaks. The -COO- peaks of SA (1629.7 cm^−1^, 1416.5 cm^−1^) remain independent, indicating no chemical bonding between the two. Therefore, SA-PO-SECs likely exhibit slight surface adsorption (weak hydrogen bonds between PO and SA hydroxyl/carboxyl groups).

In the Ca-Alg@PO-SECs spectrum, the -OH peak further broadens and shifts to a lower wavenumber (3407 cm^−1^), suggesting coordination between Ca^2+^ and the hydroxyl/carboxyl groups. Stronger -COO- double peaks appear at 1629.7 cm^−1^ and 1418.5 cm^−1^, with red-shifting compared to SA. This confirms the formation of an “egg-box” crosslinked structure between Ca^2+^ and the carboxyl groups of SA, altering the environment of the carboxylate groups. Meanwhile, the C-H stretching peak at 2927.7 cm^−1^ and the terpene structure peak at 890.6 cm^−1^ remain, showing that Ca^2+^ crosslinking significantly alters the inter-molecular hydrogen bonding and the electronic environment of the carboxyl groups, while the Pogostemon oil remains intact and effectively encapsulated in the composite structure.

In conclusion, the FTIR results show that PO is successfully encapsulated within SECs with hydrogen bonding interactions between SA and SECs at the interface. The Ca^2+^ crosslinking forms a stable three-dimensional network structure, resulting in the formation of a Ca-Alg@PO-SECs composite carrier with a structurally integrated and stable network architecture.

### 3.8. In Vitro Release Behavior

This section reports the cumulative release of PO over time in Simulated Gastric Fluid (SGF) and Simulated Intestinal Fluid (SIF). The goal is to confirm the pH-responsive nature of the Ca-Alg coating and evaluate the formulation’s potential for colonic targeting.

#### 3.8.1. Release in Simulated Gastric Fluid (SGF)

**Figure 8:**
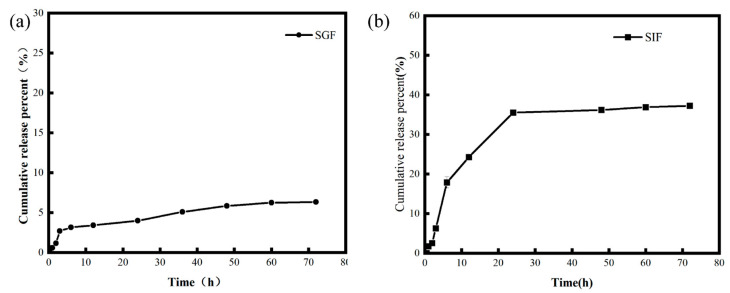
In vitro release profiles of Ca-Alg@PO-SECs. (**a**) Release profile in simulated gastric fluid (SGF). (**b**) Release profile in simulated intestinal fluid (SIF). Data are presented as mean ± SD (*n* = 3).

Figure 8b shows that PO release in Simulated Gastric Fluid (SGF) was significantly retarded. The release reached only about 10% even after 70 h. This low premature release indicates that the Ca-Alg coating remains stable and intact under acidic gastric conditions. The tightly packed calcium alginate polymer chains block SGF penetration. They prevent the early release of Pogostemon oil. This drug protects the active agent once it reaches the colon. It prevents premature absorption or inactivation in the stomach, enabling effective colonic targeting.

#### 3.8.2. Release in Simulated Intestinal Fluid (SIF)

As illustrated in Figure 8a, the cumulative release of PO from the SIF solution exhibited a rapid, sustained profile. The percentage of released PO rises sharply in the initial phase, reaching approximately 40% within the first 10 h. The release then plateaus, indicating that the majority of the encapsulated oil releases quickly and approaches a stable state. This rapid release is attributed to the pH-sensitivity of the Ca-Alg layer. At neutral pH (SIF conditions), the calcium alginate gel hydrates and swells, which increases porosity and promotes faster diffusion of PO from both the inner SECs carrier and the Ca-Alg shell. This profile successfully meets the prerequisite for a colonic-targeted system: maintaining stability in the stomach while ensuring immediate, high-concentration drug release upon reaching the intestine.

The percentage of released PO rises sharply in the initial phase, reaching approximately 40% within the first 10 h. The release then plateaus, indicating that the majority of the encapsulated oil releases quickly and approaches a stable state. The rapid release is attributed to the pH-sensitivity of the Ca-Alg layer. At neutral pH (SIF conditions), the calcium alginate gel hydrates and swells, which increases porosity and promotes faster diffusion of PO from both the inner SECs carrier and the Ca-Alg shell. Under these aqueous conditions, the release behavior is governed by matrix hydration and diffusion rather than by surface superhydrophobicity. This profile successfully meets the prerequisite for a colonic-targeted system: maintaining stability in the stomach while ensuring immediate, high-concentration drug release upon reaching the intestine.

#### 3.8.3. Summary of Release Mode

This pH-responsive drug delivery system enables precise spatiotemporal regulation of drug release through a dual-strategy mechanism. First, in the acidic gastric environment, the formulation inhibits premature release of PO, maximizing its antioxidant capacity and preventing oxidative degradation in the stomach. This stage corresponds to the adsorption and retention-dominated phase.

Then, upon reaching the neutral intestinal environment, the formulation rapidly releases a high concentration of PO, effectively targeting ROS at inflammatory sites and directly alleviating local oxidative stress damage.

This pH-responsive drug delivery system enables precise spatiotemporal regulation of drug release through a dual-strategy mechanism. First, in the acidic gastric environment, the formulation inhibits premature release of PO, maximizing its antioxidant capacity and preventing oxidative degradation in the stomach. This stage represents a release-activated process primarily controlled by Ca-Alg hydration and environmental responsiveness.

### 3.9. Mouse Ulcerative Colitis ExperimentS

This section evaluates the therapeutic effects of PO and its spore powder formulations (PO-SECs and Ca-Alg@PO-SECs) in an acute UC mouse model. The efficacy of the formulations is assessed by examining comprehensive macroscopic indicators, including daily food and water intake and crucial body weight changes, which are primary indicators of disease severity.

The therapeutic performance of PO and its spore-derived formulations was evaluated using macroscopic disease-related indicators, including body weight change and intake behavior, which are commonly used to reflect disease progression in acute UC models.

#### 3.9.1. Daily Food and Water Intake Analysis

**Figure 9:**
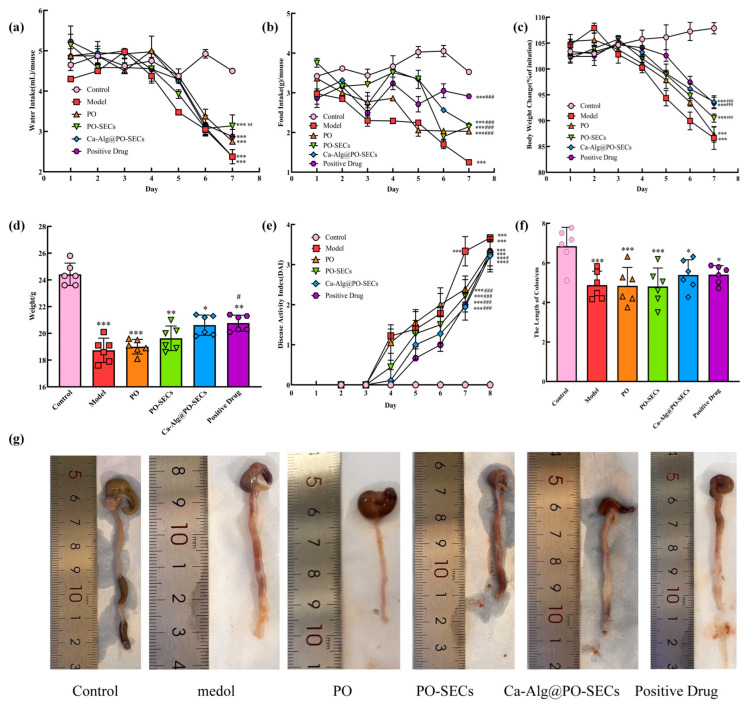
Evaluation of physiological parameters and disease progression in mice. (**a**) Daily water intake per mouse in each experimental group. (**b**) Daily food intake per mouse in each experimental group. (**c**) Daily body weight change rate per mouse in each experimental group. (**d**) Body weight per mouse in each experimental group on day 8. (*n* = 6) (**e**) Daily disease activity index (DAI) score per mouse in each experimental group. (*n* = 6) (**f**) Colon length per mouse in each experimental group on day 8. (*n* = 6) (**g**) Representative photograph of the colon from each experimental group. (Compared with the control group, * *p* < 0.05; ** *p* < 0.01; *** *p* < 0.001; compared with the model group, ^#^*p* < 0.05; ^##^ *p* < 0.01; ^###^ *p* < 0.001; no significant difference, *p* > 0.05). Data are presented as mean ± SD (*n* = 6 biological replicates). Statistical significance was determined using one-way ANOVA followed by Tukey’s multiple comparison test.

Figure 9a,b shows that daily food and water intake stayed stable in the Control group, confirming normal physiological status. In contrast, all five colitis-induced groups (Model, PO, PO-SECs, Ca-Alg@PO-SECs, Positive Drug) had a significant decline in food intake during later stages (*p* < 0.001). This confirms that the acute colitis model was successful and highlights the severe impact of colitis on feeding behavior.

The analysis of water intake yielded mixed results regarding treatment effects: The PO-SECs group had significantly higher water intake than the Model group (*p* < 0.01). This suggests a regulatory effect. It may be related to the spore powder’s high adsorption properties, which could affect behavior to ease digestive discomfort or aid absorption. Conversely, the PO, Ca-Alg@PO-SECs and Positive Drug groups did not differ significantly from the Model group in water intake. While the formulations demonstrate therapeutic effects on the disease pathology itself (as evidenced by weight changes), their impact on the specific behavioral marker of abnormal daily water intake varies significantly across delivery systems.

#### 3.9.2. Weight Change Percentage and Final Body Weight

Figure 9c shows that the Control group mice gained weight, reflecting normal growth. All five colitis-induced groups lost weight rapidly starting on Day 3. By Day 7, their weights were significantly lower than the Control group (*p* < 0.001). This confirms that acute colitis was correctly induced. Weight loss was significantly slower for the PO-SECs, Ca-Alg@PO-SECs, and Positive Drug groups compared to the Model group. These differences were seen at later stages (*p* < 0.001). These treatments effectively reduced acute colitis-induced weight loss.

Analysis of the final body weight (Figure 9d) further quantified the therapeutic efficacy: The Control group had the highest final body weight, staying within normal limits. The Model group had a much lower final body weight (*p* < 0.001). The PO group exhibited the least effective recovery; its final body weight remained significantly lower than that of the Control group (*p* < 0.001), indicating only a mild therapeutic effect. Both the PO-SECs group and the Positive Drug group improved compared to the Model group. However, their final body weights remained significantly lower than those of the Control group (*p* < 0.01). The Ca-Alg@PO-SECs group had the final body weight closest to that of the Control group. The difference was much smaller than in other treatment groups (*p* < 0.05). This shows Ca-Alg@PO-SECs best mitigated colitis-induced weight loss among all treatments.

#### 3.9.3. DAI

Figure 9e shows that the DAI in the Control group remained consistently around throughout the experiment. This indicates that the mice were healthy and showed no disease-related symptoms. In contrast, mice receiving 3.0% DSS showed a rapid increase in DAI, beginning approximately on day 3. This reached a high level by day 7. The trend confirmed the successful establishment of the colitis model. Disease activity progressively worsened over time.

From day 0 to day 3, no notable changes in DAI were observed in any group. Between days 3 and 7, mice in the PO, PO-SECs, Ca-Alg@PO-SECs, and Positive Drug groups developed varying degrees of loose stool, diarrhea, and hematochezia. Compared with the healthy Control group, mice in the DSS Model group exhibited a significant reduction in body weight (*p* < 0.001). The DAI trajectories of the treatment groups resembled those of the Model group. However, they rose at a markedly slower rate (*p* < 0.001). This indicates partial mitigation of DSS-induced disease progression.

On day 8, following the withdrawal of DSS and all treatments, only the Ca-Alg@PO-SECs and Positive Drug groups showed significant differences from the Model group (*p* < 0.05). These findings suggest that Ca-Alg@PO-SECs and the positive control drug effectively suppressed disease activity. They also reduced colitis severity.

#### 3.9.4. Colon Length

Quantitative analysis of colon length is shown in Figure 9f. Representative photographs of colons from each group are shown in Figure 9g. The Control group maintained the longest colon length, reflecting normal physiology. The Model group exhibited significantly shorter colons compared with Control (*p* < 0.001), confirming successful induction of acute colitis. Colon length in the PO and PO-SECs groups remained significantly shorter than Control (*p* < 0.001), indicating limited therapeutic effect. The Ca-Alg@PO-SECs and Positive Drug groups showed a trend toward longer colons compared with the Model group; however, no statistically significant difference was observed (*p* > 0.05), indicating only partial structural recovery toward normal levels.

#### 3.9.5. ELISA Analysis

**Figure 10:**
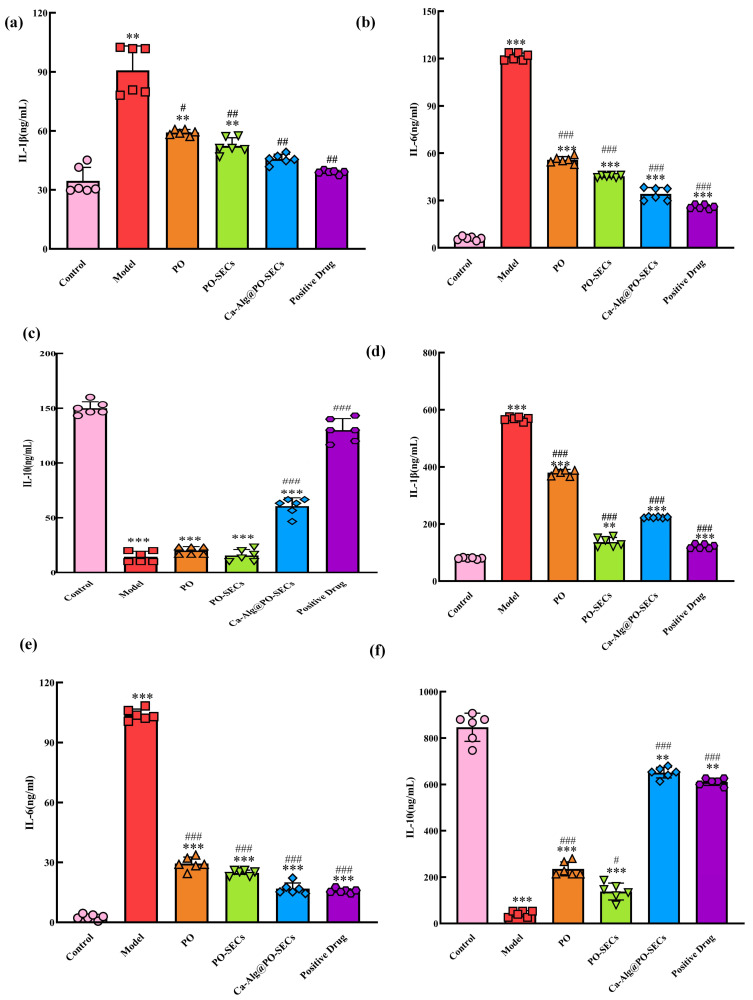
Levels of inflammatory cytokines in mice. (**a**–**c**) Concentrations of IL-1β, IL-6,and IL-10 in mouse blood. (*n* = 6) (**d**–**f**) Concentrations of IL-1β, IL-6,and IL-10 in mouse colon tissue. (*n* = 6) (Compared with the control group, ** *p* < 0.01; *** *p* < 0.001; compared with the model group, # *p* < 0.05, ## *p* < 0.01; ### *p* < 0.001). Data are presented as mean ± SD (*n* = 6 biological replicates). Statistical significance was determined using one-way ANOVA followed by Tukey’s multiple comparison test.

In Figure 10, we analyzed the cytokine profile in both blood and colon tissue to quantify the anti-inflammatory mechanisms. The pathogenesis of UC is closely tied to oxidative stress. DSS-induced disruption of the intestinal barrier causes excess ROS, worsening inflammation. While direct oxidative stress markers (such as MDA, SOD, and GSH) were not quantified in this study, it is well-established that a decrease in pro-inflammatory mediators is often associated with reduced inflammation and may reflect a lower oxidative stress status.

The Control group mice showed low levels of pro-inflammatory cytokines (IL-1β and IL-6) and high levels of the anti-inflammatory cytokine (IL-10). In contrast, the Model group displayed significant systemic and local inflammation, defined by Elevated levels of IL-1β (blood: *p* < 0.01; colon: *p* < 0.001) and IL-6 (blood: *p* < 0.001; colon: *p* < 0.001) and Reduced levels of IL-10 (blood: *p* < 0.001; colon: *p* < 0.001).

All treatment groups (PO, PO-SECs, Ca-Alg@PO-SECs, and Positive Drug) significantly reduced IL-1β (*p* < 0.05) and IL-6 (*p* < 0.001) levels compared with the Model group, suggesting an effective suppression of pro-inflammatory cytokine production.

Ca-Alg@PO-SECs and the Positive Drug group achieved blood IL-1β levels statistically similar to the Control group (no significant difference). Furthermore, all treatment groups showed strong inhibition of IL-1β in colon tissue (*p* < 0.001 compared to the Model group). All treatment groups significantly reduced IL-6 secretion (*p* < 0.001 compared to the Model group).

For the anti-inflammatory cytokine IL-10, differences in delivery system efficacy were apparent: Only the Ca-Alg@PO-SECs and Positive Drug groups significantly increased blood IL-10 levels (*p* < 0.001 compared to the Model group), partially restoring systemic anti-inflammatory activity. The PO group significantly elevated colon IL-10 levels (*p* < 0.001), whereas the PO-SECs group produced a comparatively weaker effect.

In summary, the Ca-Alg@PO-SECs formulation and the Positive Drug group demonstrated the most effective overall anti-inflammatory effects. Both treatments suppressed production of pro-inflammatory cytokines (IL-1β and IL-6) in blood and colon tissue and promoted the critical secretion of the anti-inflammatory cytokine IL-10. Given the close crosstalk between inflammation and oxidative stress in UC, these results suggest a potential association with alleviated oxidative stress, which may contribute to the reduction in ROS-induced mucosal damage. Future studies incorporating direct antioxidant enzyme assays will be necessary to fully elucidate this specific mechanism.

#### 3.9.6. Organ Coefficient

**Figure 11:**
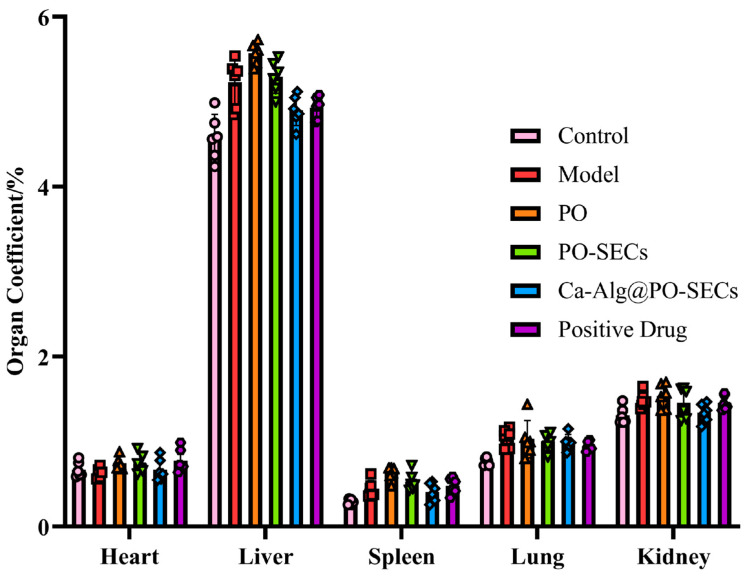
Organ indices of major organs, including liver, spleen, kidney, heart, lung, and thymus, in each experimental group. Data are presented as mean ± SD (*n* = 6 biological replicates).

In Figure 11, the Model group mice showed an increased relative organ weight (organ coefficient) for liver, spleen, lungs, kidneys, and other organs, indicating that multiple organs were affected in the pathological state. The treatments of PO, PO-SECs, Ca-Alg@PO-SECs, and Positive Drug all reversed this abnormality to varying degrees, with Ca-Alg@PO-SECs and Positive Drug showing the most prominent improvements. The effect on the heart was minimal across all experimental groups.

## 4. Conclusions

In this study, we developed a colon-targeted microsphere system, Ca-Alg@PO-SECs, by integrating a hydrophobic sporopollenin exine capsule (SEC) core with a pH-sensitive calcium alginate (Ca-Alg) shell. This multi-layered design enhanced the physicochemical stability of Pogostemon oil (PO). It also enabled controlled, pH-responsive release under simulated gastrointestinal conditions.

The engineered SECs showed high structural integrity and greater surface hydrophobicity. This facilitated efficient PO encapsulation and allowed a high loading capacity through optimized selective adsorption. The outer Ca-Alg shell provided a critical secondary barrier, further improving the stability of the encapsulated PO against thermal and photodegradation, which is beneficial for preserving the bioactivity of volatile essential oils during delivery.

In a DSS-induced acute ulcerative colitis mouse model, Ca-Alg@PO-SECs showed more pronounced therapeutic effects than free PO or PO-SECs. Improvements included recovery of body weight and colon length, reduction in the disease activity index (DAI), and restoration of colonic histological architecture. In addition, the formulation modulated systemic inflammatory cytokine profiles by suppressing pro-inflammatory mediators. However, the study did not assess potential sex-dependent differences in disease progression and therapeutic response—an acknowledge limitation. However, potential sex-dependent differences in disease progression and therapeutic response were not assessed and are acknowledged as a limitation.

Although we did not directly measure oxidative stress in this study, the enhanced physicochemical stability of PO and the observed modulation of inflammatory cytokines suggest that Ca-Alg@PO-SECs may indirectly affect oxidative processes linked to inflammation during colonic transit. Further studies should include direct measurements of oxidative stress biomarkers to clarify these mechanisms. No clinical trials have been reported to date. Therefore, the long-term safety and immunogenicity of SECs in humans remain unknown and require dedicated investigation before clinical application.

## Figures and Tables

**Table 1:** Disease Activity Index (DAI) scores of mice in each experimental group over the study period.

DAI	Weight Loss Percentage (%)	Fecal Consistency	Fecal Color
0	No change	Normal	Normal
1	1~5		
2	6~10	Loose	Black stools
3	10~20		
4	>20	Diarrhea	Bloody stool

**Table 2:** Optimization of adsorption conditions for PO using SECs under different adsorption methods.

Adsorption Method	Optimal SECs Dosage (g/L)	Optimal Adsorption Time (h)	Optimal Pogostemon Oil Concentration (g/L)	Core Differences (Compared to Other Methods)
Passive Adsorption	8	3	20	Fastest adsorption equilibrium (3 h to stabilize), very low efficiency at low concentration (only 6.62% at 2 g/L).
Vacuum Adsorption	8	4	20	Slower equilibrium speed (4 h to stabilize), higher efficiency at low concentration (20.49% at 2 g/L) than passive adsorption.
Selective Adsorption	8	2	40	Highest efficiency limit (69.11% at 40 g/L), significantly higher adsorption capacity (27.64 g/g at 40 g/L) than the other two methods.

**Table 3:** Stability of PO, PO-SECs, and Ca-Alg@PO-SECs under different stress conditions (*n* = 3).

Stability Experiment	Sample	Weight Mass (%)			Significant Differences
		Day 0	Day 5	Day 10	
High-Temperature	PO	-	−77.5035 ± 0.143	−90.3051 ± 0.3036	***
	PO-SECs	-	−57.1999 ± 0.198	−60.2471 ± 0.2447	***
	Ca-Alg@PO-SECs	-	−21.895 ± 0.037	−30.1331 ± 0.1089	
High-Humidity	PO	-	−33.4615 ± 0.3928	−66.3021 ± 0.1522	***
	PO-SECs	-	−14.5594 ± 0.3484	−32.48412 ± 0.1878	***
	Ca-Alg@PO-SECs	-	29.8286 ± 0.0342	40.13689 ± 0.1793	
Strong Light	PO	-	−14.6478 ± 0.3544	−61.6328 ± 0.2729	***
	PO-SECs	-	−8.6535 ± 0.2184	−32.3811 ± 0.2316	***
	Ca-Alg@PO-SECs	-	−1.9352 ± 0.0741	−4.8639 ± 0.2001	

Note: Data are presented as mean ± SD (*n* = 3). Negative values indicate mass loss due to volatilization or degradation, while positive values represent moisture absorption. Statistical significance was determined using one-way ANOVA followed by Tukey’s multiple comparison test. Asterisks (***) denote significant differences (*p* < 0.001) between the indicated group and the Ca-Alg@PO-SECs group under the same conditions.

## Data Availability

The original contributions presented in this study are included in the article/Supplementary Material. Further inquiries can be directed to the corresponding authors.

## Supplementary Materials

The following supporting information can be downloaded at https://www.mdpi.com/article/10.3390/antiox15010116/s1, Figure S1. SEM images showing the morphological changes of pollen grains after prolonged acidolysis treatment. Figure S2. Statistical analysis of the significant differences in water contact angles between UT-SFP and AT-SFP. Figure S3. Formation of marble-like water droplets on the surface of treated sporopollenin-based materials. Figure S4. Statistical analysis of weight mass changes of PO, PO-SECs, and Ca-Alg@PO-SECs under high-temperature, high-humidity, and strong-light conditions. Video S1. Water adsorption behavior of sporopollenin-based carriers visualized using methylene blue staining. Video S2. Adsorption of soybean oil by sporopollenin-based carriers visualized using Sudan III staining. Video S3. Adsorption behavior of *Pogostemon cablin* essential oil (PO) by sporopollenin-based carriers.
