# Supplementary figures and images for: Engineered Sporopollenin Exine Capsules for Colon-Targeted Delivery and Antioxidant Therapy of Pogostemon Oil in Ulcerative Colitis
Source: Antioxidants (Basel). 2026 Jan 16;15(1):116. doi: 10.3390/antiox15010116 (PMC12837650; doi:10.3390/antiox15010116)

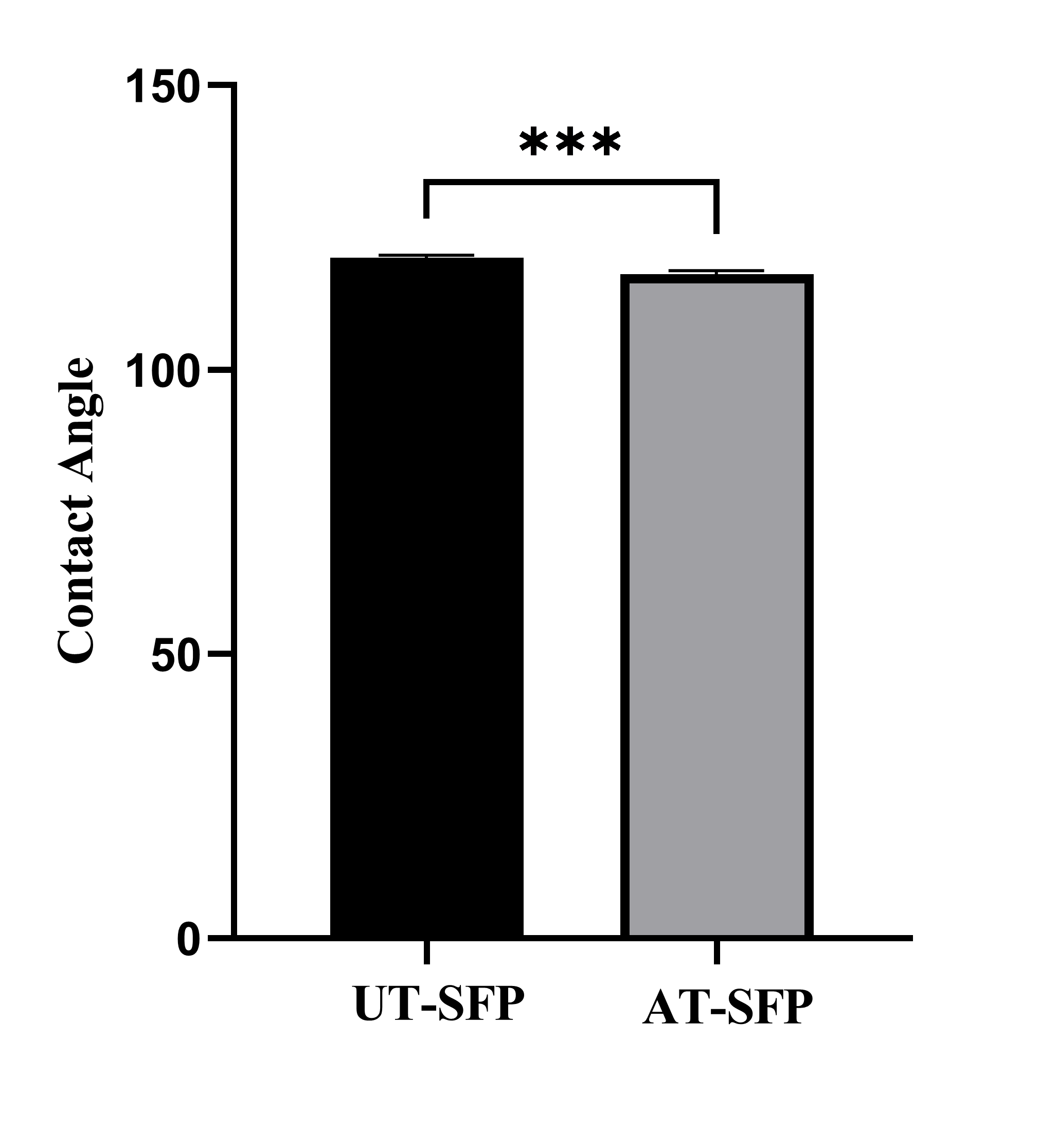

Supplement: Supplementary file 1 [file antioxidants-15-00116-s001.zip › Figure S2.tif]

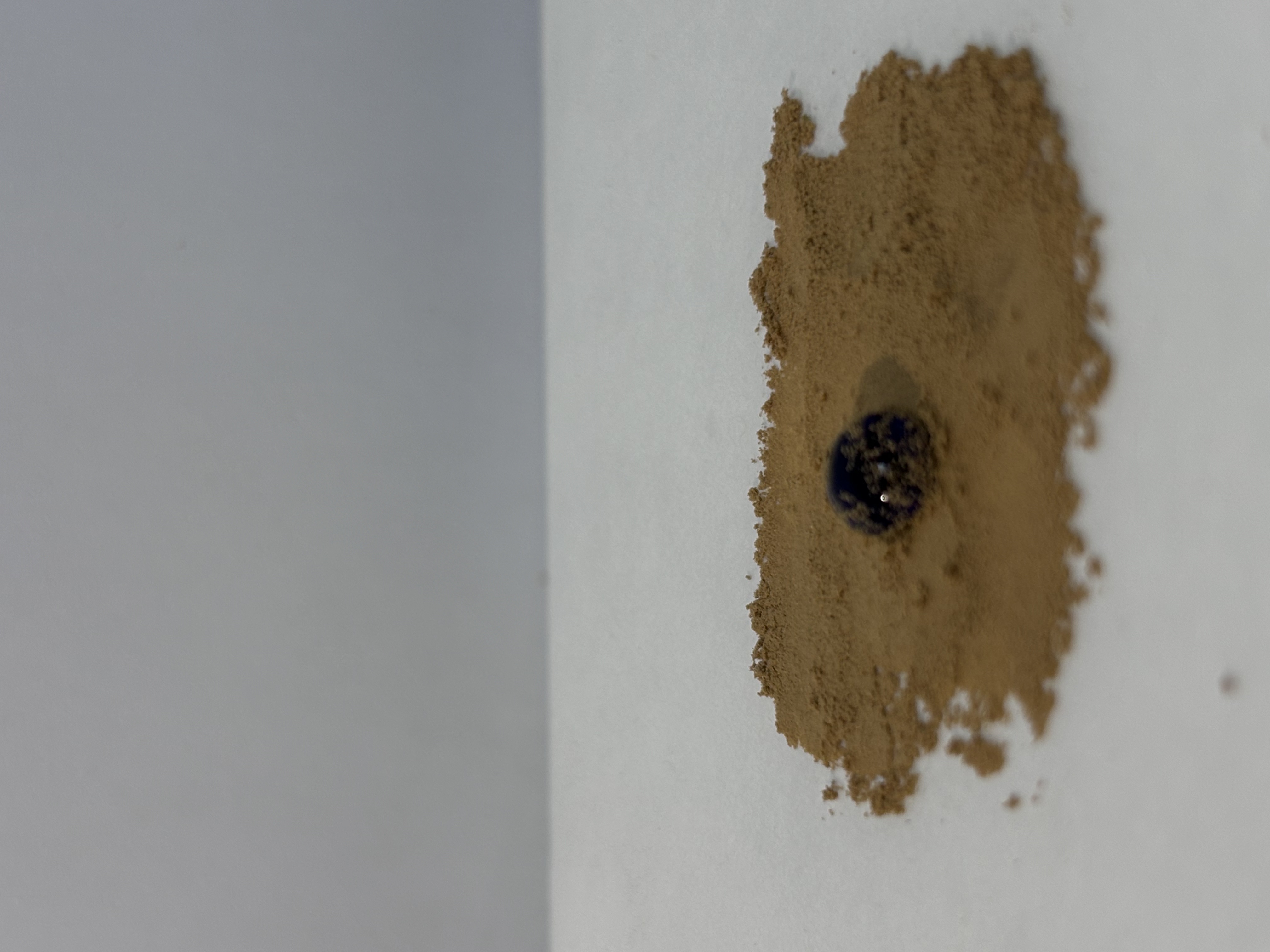

Supplement: Supplementary file 1 [file antioxidants-15-00116-s001.zip › Figure S3.jpg]

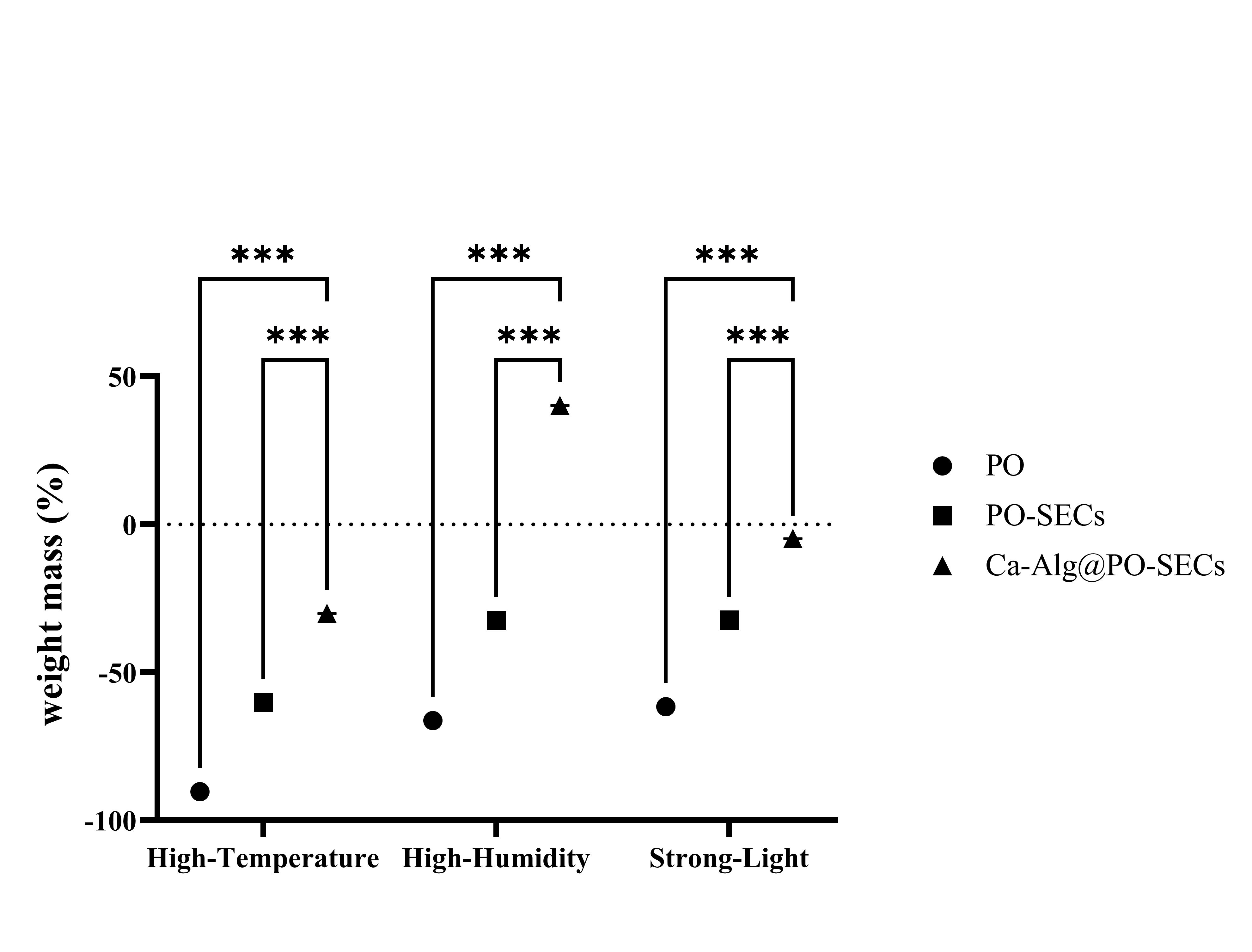

Supplement: Supplementary file 1 [file antioxidants-15-00116-s001.zip › Figure S4.tif]
